# Outer Membrane Vesicles Derived from Oral Bacteria Act as a Dagger in Host Immunity: Insight Tips from Local Diseases to Systemic Effects

**DOI:** 10.7150/ijbs.121559

**Published:** 2025-10-01

**Authors:** Xinyue Zhang, Jianing Wu, Zhe Zhang, Chengcheng Liu, Jing Xie

**Affiliations:** 1 State Key Laboratory of Oral Diseases & National Center for Stomatology & National Clinical Research Center for Oral Diseases, West China Hospital of Stomatology, Sichuan University, Chengdu 610041, Sichuan, China.; 2 State Key Laboratory of Oral Diseases & National Center for Stomatology & National Clinical Research Center for Oral Diseases, Department of Periodontics, West China Hospital of Stomatology, Sichuan University, Chengdu 610041, Sichuan, China.; 3 MOE Joint International Research Laboratory of Pancreatic Diseases, Department of Hepatobiliary and Pancreatic Surgery, the First Affiliated Hospital, Zhejiang University School of Medicine, Hangzhou 310003, Zhejiang, China.

**Keywords:** outer membrane vesicles, oral flora, periodontitis, oral squamous cell carcinoma

## Abstract

Outer membrane vesicles (OMVs) are secreted by gram-negative bacteria and are genetically and environmentally regulated. The contents of OMVs are derived from the outer membrane and periplasm of bacteria, that can act as virulence factors to attack host cells. In periodontitis, the OMVs of *Porphyromonas gingivalis* and other important periodontal pathogens can destroy the host structure, induce host immune responses, and promote periodontitis progression. In oral squamous cell carcinoma (OSCC), OMVs accelerate cancer spread and metastasis by regulating the gene expression of tumour cells. In addition to their role in oral diseases, OMVs can spread from the oral cavity to the whole body, thereby participating in the development of many diseases, including circulatory diseases, endocrine diseases, autoimmune diseases, and neurologic diseases. In this review, we introduce the biogenesis, basic structure, and roles of OMVs and comprehensively summarize the biological characteristics of OMVs from various oral bacteria. In addition, we describe the impact of OMVs on oral diseases as well as systemic health and emphasize their therapeutic potential as drug targets, antigens, and immune adjuvants for application in periodontitis and OSCC. Finally, we discuss in depth the future research directions, application prospects, and challenges of OMVs.

## 1. Introduction

Extracellular vesicles (EVs) are spherical nanoparticles with a double-layered lipid membrane that can package and transport cytoplasmic cargoes.^1^ They have been detected in various organisms, such as eukaryotes, bacteria, and archaea. In vertebrate cells, EVs are called exosomes, which are produced by sequential invagination of the plasma membrane and have an endosomal origin.^2^ In bacteria, EVs are called bacterial extracellular vesicles (BEVs) 3, which can be further divided into four categories: outer membrane vesicles (OMVs), outer inner membrane vesicles (OIMVs), tube-shaped membranous structures (TSMSs), and cytoplasmic membrane vesicles (CMVs).^4^ Among these subtypes, OMVs, which were first identified as lysine-restricted secretions of *Escherichia coli* in 1965 5 and subsequently found to be derived from the bacterial outer membrane (OM) in 1966 6, have received the most attention. They are squeezed from the OM and contain the periplasm of gram-negative bacteria.^3^, ^7^ This process is regulated by intrinsic genes when stimulated by environmental chemical or physical factors.^4^ OMVs perform multiple functions via cooperation or competition with bacteria in various complex microenvironments.^8^, ^9^ Depending on their parental bacterial categories and the stage of the diseases in the host, OMVs can either benefit host health or participate in pathogenesis, indicating their vital bilateral potential.^10^, ^11^ The importance of OMVs is becoming increasingly prominent with continuous research efforts. Recently, OMVs in the oral cavity have gotten much attention. They have been reported to perform in structural damage and inflammatory dysregulation in the periodontal tissues and the metastasis of oral cancer.^12^, ^13^ Notably, OMVs also have the unique ability to spread diseases from the oral cavity to the whole body, which the other EVs cannot achieve. Meanwhile, their therapeutic potential, involving acting as vaccines, drug carriers, and drug targets, also possess great application prospects. This review aims to illuminate the pathogenic role and therapeutic potential of OMVs in oral diseases, particularly periodontitis and oral squamous cell carcinoma (OSCC), as well as their broad implications for health, with the aim of more thorough research into this field and the development of more efficacious, safer treatments targeting OMVs.

## 2. Conspectus of OMVs

**Figure 1:**
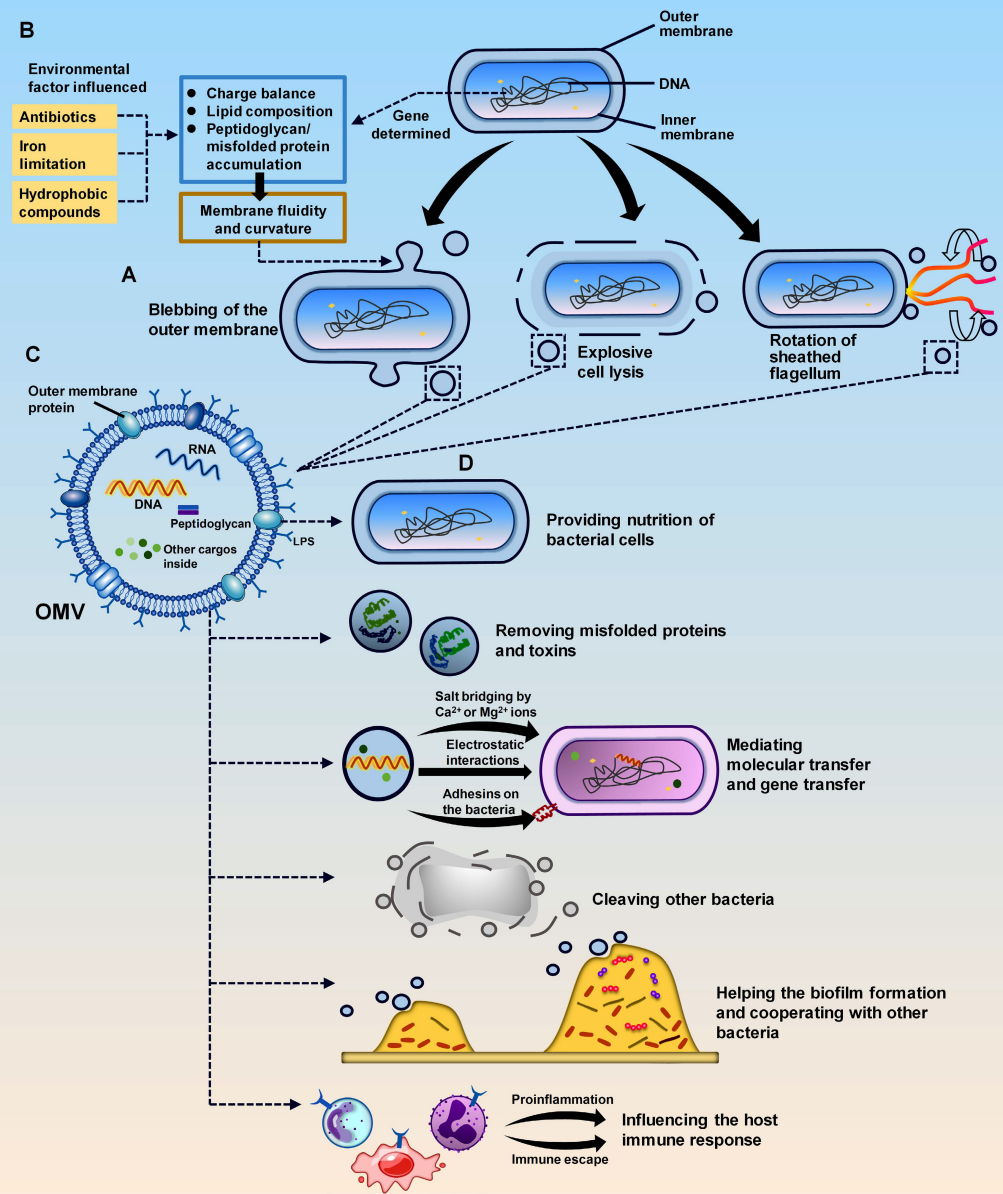
** Biogenesis, basic structure, and roles of OMVs.** (**A**). The biogenesis of OMVs is genetically determined by bacteria and influenced by the environment. The environmental factors include antibiotics, iron limitation, and hydrophobic compounds. Together with genes, these factors affect the charge balance, lipid composition, and peptidoglycan/misfolded protein accumulation of the bacterial outer membrane. These changes in membrane fluidity and curvature ultimately lead to the biogenesis of OMVs. (**B**). OMVs are produced in three ways: blebbing, explosive cell lysis, and the unique rotation of sheathed flagella in *V. fischeri*. (**C**). OMVs are composed of lipids such as LPS and OM proteins on the membrane and peptidoglycan, nucleic acid, and other cargos inside the vesicle. (**D**). The main roles of OMVs include providing nutrients to bacterial cells, mediating molecular transfer and gene transfer, causing cleavage of other bacteria, and eliciting the host immune response.

Due to bacterial diversity, the biogenetic processes of OMVs differ among bacteria. Overall, there are two fundamental methods: blebbing of the OM and explosive cell lysis (**Figure 1A**).^4^, ^14^, ^15^ Both modes of OMV generation are influenced by genetic factors and environmental conditions (**Figure 1B**).^4^ OM blebbing is triggered mainly by genes and is partially affected by environmental factors. Genes regulate membrane curvature and fluidity through intracellular events, including cross-linkage between peptidoglycan and the OM, accumulation of periplasmic peptidoglycan fragments or misfolded proteins, and composition formation of the OM.^7^, ^16^, ^17^ Environmental conditions, such as antibiotics, iron limitations, and hydrophobic compounds, also disturb the membrane and impact the blebbing process and content of OMVs.^16^, ^18^-^20^ Notably, some special bacterial structures can also trigger OM blebbing. In *Vibrio fischeri*, OMVs can be produced by the rotating flagellum, which is surrounded by a sheath structure derived from the OM (**Figure 1B**).^21^ In the case of explosive cell lysis, it is triggered by endolysins of double-stranded DNA phages. Endolysins rely on small hydrophobic proteins called holins to enter peptidoglycans and trigger cell lysis, producing OMVs while releasing new phage particles.^22^ Environmental conditions, such as the presence of DNA-damaging agents and peptidoglycan-degrading enzymes, can facilitate this process.^22^ OMV biogenesis may lead to a small percentage of bacterial cell death but is generally favourable for the survival of the whole bacterial community. The produced OMVs can neutralize environmental factors that are harmful to bacterial survival in the environment and protect the remaining bacterial population.^4^, ^23^

OMVs are spherical vesicles composed of a membrane surface and internally wrapped cargos.^3^ Since they are encapsulated only by the bacterial OM and the inner membrane is not involved, OMVs encompass molecules from the OM as well as the periplasm.^3^ These molecules are usually mature biomolecules, including proteins, lipids, and nucleic acids, which serve as both structural and virulent components of OMVs (**Figure 1C**).^19^ Proteins, such as gingipains in *Porphyromonas gingivalis* OMVs 24 and leukotoxin A (LtxA) and cytolethal distending toxin (CDT) in *c* OMVs 25, 26, play crucial roles in bacterial toxicity. Lipopolysaccharide (LPS) is an important lipid on the surface of OMVs and originates from the OM of gram-negative bacteria. It participates in the formation of pathogen-associated molecular patterns (PAMPs) to interact with the host.^27^-^29^ LPS and proteases may represent heat-stable or heat-unstable components in OMVs and are involved in interactions with the host.^30^ Nucleic acids in OMVs can act as signalling molecules to mediate intercellular communication and gene transfer.^30^-^33^

Compared with bacteria, OMVs are more invasive, contain higher concentrations of virulence factors as well as endow some of them with new forms and functions, and spread faster and farther.^34^, ^35^ For example, *P. gingivalis* OMVs enter oral cells such as human oral keratinocytes (HOKs) and human gingival fibroblasts (HGFs) more easily than the original bacteria do.^36^-^40^ They can also accelerate the aggregation of a large class of bacteria and facilitate the entry of some viruses into oral cells.^41^, ^42^

## 3. Functions of OMVs from various bacteria

OMVs function via two main mechanisms. First, OMVs burst near target cells and shed their contents at very high localized concentrations.^19^, ^43^ Second, OMVs bind to the surface of target cells and undergo proximal cleavage, phagocytosis, internalization, or membrane fusion as a whole delivery of contents.^19^, ^43^-^46^ Both mechanisms enable the intercellular cargo transfer of OMVs and horizontal gene transfer among bacterial species. For example, *Acinetobacter baylyi* OMVs can transfer small DNA fragments to* E. coli.*^47^

OMVs exhibit specific functions in interactions with bacteria and the host (**Figure 1D**).^48^ In terms of communication with bacteria, OMVs protect parental bacteria, cooperate and compete with other bacteria, and promote biofilm formation. In addition, OMVs play a trophic role in parental bacteria. They package cargoes such as proteins and enzymes to promote nutrient uptake and balance in the bacterial community.^49^, ^50^ OMVs also increase bacterial resistance by removing misfolded proteins and toxins from bacteria. For other bacteria, OMVs participate in killing competing microorganisms, acting as protectors of the parental bacteria.^51^ This recognition mechanism is mediated by peptidoglycan hydrolases, which do not cleave the peptidoglycan layer when they are identical to those of the target strains but degrade the cell wall and kill the target cells when they are different.^52^ Thus, OMVs have the potential to treat infections caused by other bacteria.^53^, ^54^ In addition to their competitive effects, OMVs can synergize with other bacteria that share the same pathogenicity. For example, OMVs from the periodontal pathogen *P. gingivalis* can inhibit the bactericidal activity of serum against another periodontal pathogen, *Capnocytophaga ochracea.*^55^, ^56^ OMVs also engage in biofilm formation under pressure and mediate the delivery of growth factors and extracellular matrix components in biofilms.^57^-^59^ OMV-mediated release of extracellular polysaccharides enhances bacterial coaggregation in biofilms.^60^

In OMV-host interactions, OMVs can promote pathogenesis through their virulence factors and distant diffusion ability. However, not all OMVs are harmful to the host, and some are actually beneficial to human health. OMVs from probiotics can show therapeutic potential.^61^-^63^ Moreover, OMVs from some pathogens can also act as drug targets, drug carriers, and vaccines to prevent and treat diseases. This is decided by the stage of diseases that OMVs occur. Before the occurrence of diseases, OMVs from pathogens can be applied in disease prevention. During the progression of the diseases, OMVs from pathogens, as an important carrier of virulence factors, promote the disease process. When treating diseases, these OMVs may serve as a target for drug therapy. And OMVs from probiotics may play a therapeutic role throughout the entire process of disease. Thus, given their wide range of implications, OMVs represent current research directions in both pathogenesis and therapeutic strategies.

**Figure 2:**
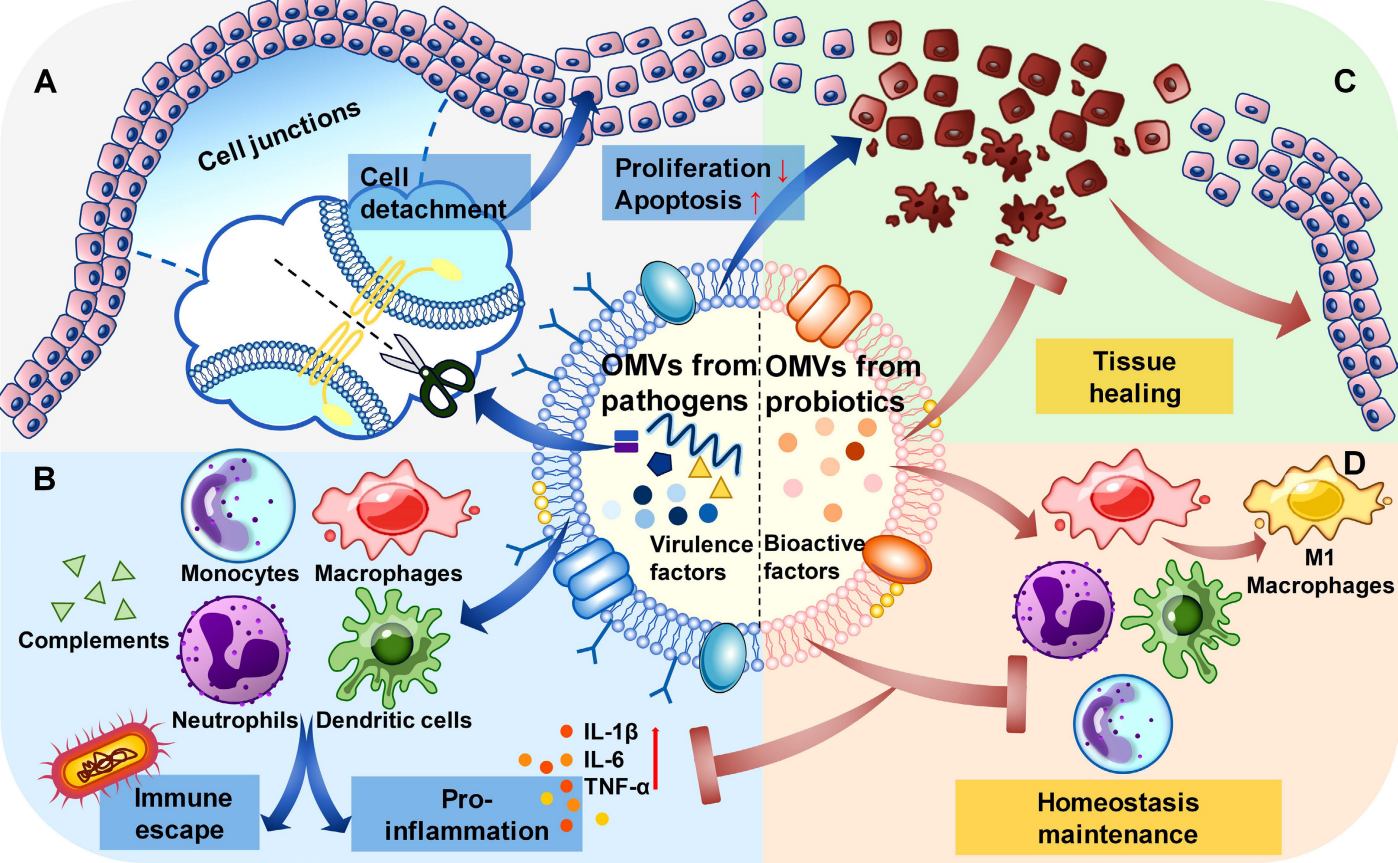
** Functions of different OMVs in the epithelial cells and immune system.** (**A**). In the epithelial cells, OMVs from pathogens destroy the cell junctions to cause cell detachment, promote cell apoptosis and inhibit cell proliferation. (**B**). In the immune system, OMVs from pathogens interact with immune cells and consume complements, leading to proinflammation in general and the immune escape of specific bacteria. (**C**). In the epithelial cells, OMVs from probiotics can inhibit the structural destruction caused by pathogens and promote tissue healing. (**D**). In the immune system, OMVs from probiotics adjust the state of immune cells to maintain homeostasis.

OMVs from different bacteria function in different mechanisms. For OMVs from pathogens, they tend to invade and spread in the epithelial cells by destroying the cell junctions, inhibiting proliferation, and inducing apoptosis of the epithelial cells (**Figure 2A**). These processes may be affected by the type of parental bacteria and the competitive relationship with the other bacteria. The functions of OMVs from pathogens in the immune system include two aspects: proinflammation and immune escape. OMVs from pathogens enhance the immune response through their virulence factors in general, while for their parental bacteria and those with the same pathogenic goal, they can promote immune escape by consuming complements in the serum and pre-treating the immune cells (**Figure 2B**). For OMVs from probiotics, they can promote the healing process in the epithelial tissues infected by pathogenic bacteria (**Figure 2C**).^64^ And their role in the immune system may be to maintain homeostasis, regulating inflammatory responses during the immune dysregulation, and promoting immune responses when the immune system is inhibited (**Figure 2D**).^65^-^67^

OMVs can contribute to destruction of the host structure, promote inflammation, and mediate the spread of diseases.^59^ For structural destruction, OMVs invade the host through virulence factors and act as a bridge to increase the adhesion of bacteria to host cells. OMVs of *Treponema denticola* can disrupt tight junction proteins between epithelial cells through the protease dentilisin, causing damage.^68^ OMVs strongly influence the host immune system to promote inflammation and are involved in a variety of inflammatory diseases, including periodontitis, gastrointestinal inflammation, pulmonary inflammation, and sepsis.^69^-^72^ In inflammation, OMVs promote the secretion of inflammatory factors from not only immune cells but also nonimmune cells, such as gingival epithelial cells, by activating multiple signalling pathways.^73^ In addition to mediating short-range intercellular communication, OMVs spread throughout the body rather than being confined to the primary lesion.^74^ Due to their small size and high concentration of virulence factors, OMVs can be transported over long distances within the host, thereby exerting systemic effects. For example, *P. gingivalis* OMVs can induce vascular calcification, trigger rheumatoid arthritis (RA), and enter the central nervous system (CNS) to promote Alzheimer's disease (AD).^75^-^77^ *Fusobacterium nucleatum* OMVs can even accelerate the lung metastasis of oral cancer.^12^

Probiotic-derived OMVs can package dioxide nanozymes for the treatment of inflammatory bowel disease with high safety and efficiency.^10^ The therapeutic potential of OMVs from pathogenic bacteria is manifested mainly as targets of antibacterial drugs and as immune stimulators. OMVs from the periodontal pathogen *P. gingivalis*, for example, represent the target of the antibacterial drugs N-hexane-extracted fennel (HEF) and curcumin, thereby participating in their anti-inflammatory effects.^78^, ^79^ OMVs, which have high antigenicity, also function as immune stimulators. In the prevention of periodontitis,* P. gingivalis* OMVs can induce a strong immune response in the blood and saliva.^80^-^82^ Moreover, since OMVs display good biocompatibility and specificity, they are employed as carriers for antigens and drugs. Bioengineered OMVs with different tumour antigens can inhibit the metastasis of lung melanoma and suppress the growth of subcutaneous colorectal cancer 83, and they can also deliver small interfering RNAs (siRNAs) as drugs to kill cancer cells in a cell-specific manner.^84^

As inappropriate antibiotic use and drug-resistant bacteria present enormous challenges to conventional antibiotic therapy, developing more effective antimicrobial methods is vital. OMVs are promising candidates because they display antimicrobial activity and undermine bacterial resistance by stimulating host cell immunity. 85-87 OMVs also possess the capacity for disease prevention and cancer treatment as immune stimulators and carriers. After oral administration, natural OMVs spread rapidly to induce an immune response but are unsuitable for clinical use because of their uncontrolled toxicity and inaccurate targeting ability.^87^ Therefore, natural OMVs should be surface modified and express exogenous proteins, which will reduce their toxicity and increase their biocompatibility and specificity.^88^ By exerting a wide range of immunostimulatory effects, OMVs show great potential in vaccine development.^80^, ^81^ They can be loaded with different tumour antigens to strongly activate antitumour immunity with few adverse reactions.^83^, ^89^, ^90^ Appropriate OMV stimulation can even enhance immunity against other diseases 91, and OMV-targeting drugs are critical for the control of gram-negative bacterial infections. Owing to their high therapeutic potential, OMVs might open new avenues for the treatment of several diseases.

The bacterial community in the oral cavity is one of the most complex known. More than 700 bacterial species routinely colonize the human oral cavity. OMVs have been identified as important vesicles of many oral bacteria. The involvement of OMVs derived from oral bacteria in influencing host innate and adaptive immunity has been shown.

### 3.1 *P. gingivalis* OMVs

**Figure 3:**
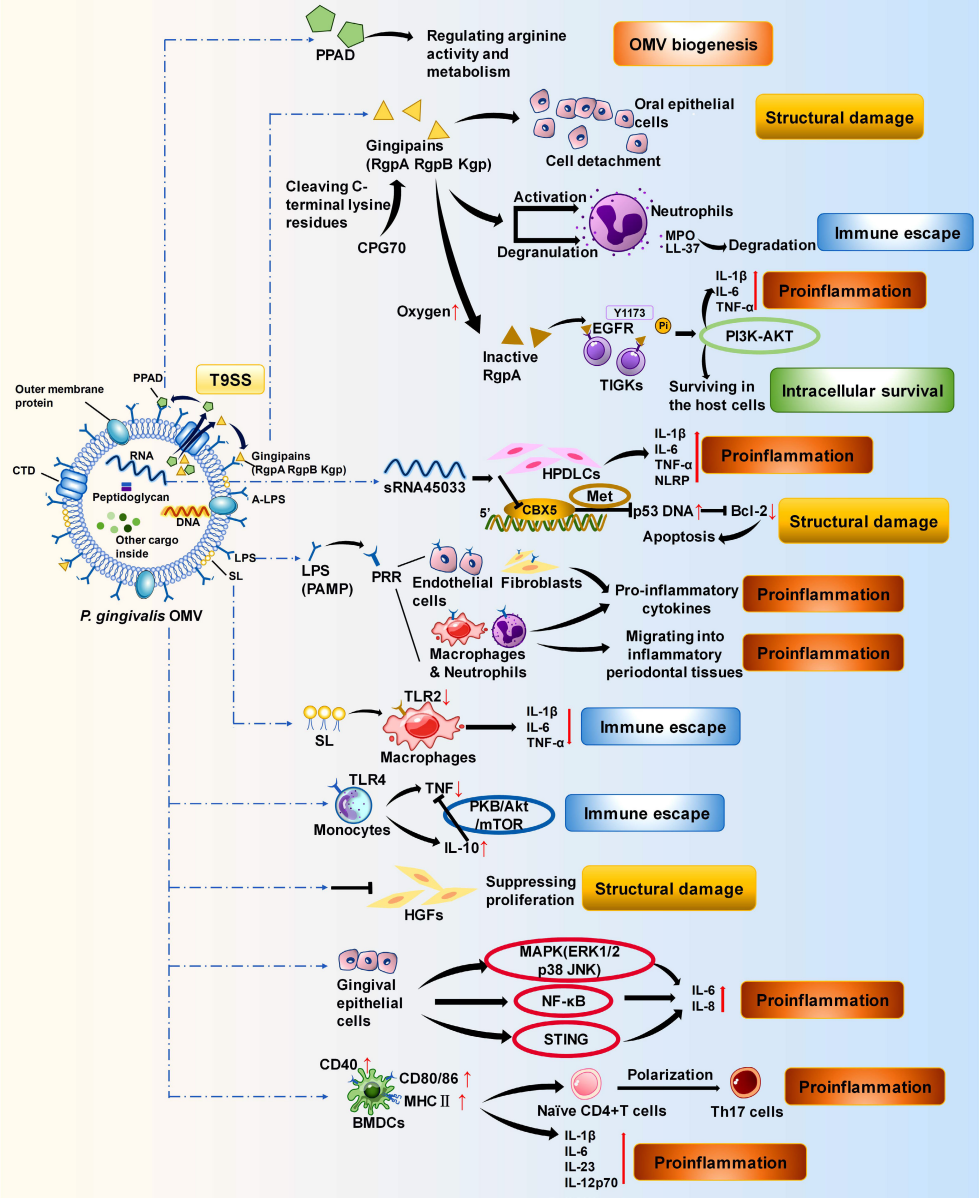
** Functions of *P. gingivalis* OMVs in promoting periodontitis.** *P. gingivalis* OMVs and their inner contents, including PPAD, gingipains, sRNA, LPS, SL, and some unknown virulence components, can promote the progression of periodontitis. Their functions include facilitating OMV biogenesis, facilitating immune escape and the intracellular survival of *P. gingivalis*, damaging the periodontal structure and promoting inflammation in various host cells through different pathways.

*P. gingivalis* OMVs can serve as immune evasion tools for bacteria to escape immune attack. They retain antigenic determinants with strong antigenicity to bind to and deplete antibodies and complements as decoys, allowing bacteria to escape immune attack during infection.^38^ In addition to passively eliminating immune factors, *P. gingivalis* OMVs also actively regulate host immunity.^92^ Due to their smaller size, *P. gingivalis* OMVs spread faster and are exposed to the immune system earlier than bacteria are. When monocytes are present, *P. gingivalis* OMVs act as gingival immune response modulators to regulate their immunocompetence and induce immune tolerance. When *P. gingivalis* restimulates the host later, monocytes prestimulated with *P. gingivalis* OMVs fail to secrete tumour necrosis factor (TNF) to respond (**Figure 3**).^92^ This may represent an immune escape strategy for *P. gingivalis*. *P. gingivalis* OMVs can also promote the maturation of bone marrow-derived dendritic cells (BMDCs) and their secretion of proinflammatory factors, and *P. gingivalis* OMV-activated BMDCs can induce Th17 polarization of naïve CD4^+^ T cells (**Figure 3**).^93^, ^94^

*P. gingivalis* OMVs contain various virulence factors of *P. gingivalis*, such as proteases, msRNAs, and lipids. These virulence factors are powerful weapons used by OMVs to disrupt the host immune response. Gingipains are cysteine proteases that are essential for the virulence of *P. gingivalis* OMVs. They are composed of three subfractions: Arg-gingipain (Rgp) A, RgpB, and Lys-gingipain (Kgp).^95^ The levels of these proteins in *P. gingivalis* OMVs are much greater than those in the parental bacteria, making *P. gingivalis* OMVs more pathogenic and invasive, which is due mainly to the type IX secretion system (T9SS) and anionic lipopolysaccharide (A-LPS) mechanisms.^96^-^100^ Gingipains in the periplasm are recruited and secreted across the OM by the T9SS via its structurally conserved C-terminal structural domain (CTD).^101^ Then, the gingipains are covalently linked to A-LPS on the OM of bacteria to build a toxic shell.^97^, ^98^ OMVs are derived from the OM and thus obtain gingipains through A-LPS. As A-LPS is more abundant on OMVs than on OMs, *P. gingivalis* OMVs are more enriched in gingipains.^96^, ^97^, ^99^ OMV-bound gingipains from *P. gingivalis* may present a “neutrophil deceptive strategy”. They explicitly activate neutrophil degranulation.^102^ Instead of being internalized by macrophages or epithelial cells, they are located on the surface of neutrophils and degrade bactericidal molecules such as the particulate enzyme myeloperoxidase (MPO) and the highly potent particulate-derived cationic antimicrobial peptide (CAMP) LL-37, undermining the host's ability to sterilize.^102^-^106^ This process can be a conserved neutrophil deceptive strategy that protects *P. gingivalis* from being engulfed by inflammatory cells, helps them colonize gingival crevices, and ultimately exacerbates periodontitis (**Figure 3**).^102^ Peptidylarginine deiminase (PPAD) is another protease cargo of the T9SS and plays an essential role in *P. gingivalis* OMVs.^101^ PPAD activity (citrullination) restricts biofilm formation and promotes surface translocation, participating in the biogenesis of *P. gingivalis* OMVs and their surface attachment.^41^, ^107^

MicroRNA-sized small RNAs (msRNAs) in *P. gingivalis* OMVs have the potential to promote host cell apoptosis and accelerate inflammation.^108^ In an *in vitro* experiment in which human periodontal ligament cells (HPDLCs) were cocultured with *P. gingivalis* OMVs, OMVs were taken up and endocytosed by HPDLCs.^108^ The msRNA sRNA45033 in *P. gingivalis* OMVs inhibited the apoptosis-related gene Chromobox 5 (CBX5) and influenced the p53/B-cell lymphoma-2 (Bcl-2) axis, indirectly facilitating the apoptosis of HPDLCs.^108^ Moreover, through promoting the release of proinflammatory factors and the production of pyrin domain containing 3 (NLRP3), sRNA45033 can also aggravate inflammation and promote pyroptosis in HPDLCs (**Figure 3**).^108^, ^109^

Lipids are also important virulence factors in *P. gingivalis* OMVs. As a component of PAMPs, LPS in *P. gingivalis* OMVs interacts with pattern recognition receptors (PRRs) on fibroblasts, epithelial cells, and macrophages, causing chronic inflammatory dysregulation.^110^, ^111^ LPS from *Tannerella forsythia* OMVs and *T. denticola* OMVs is also involved, but neither elicits a PRR response as intense as that induced by LPS from *P. gingivalis* OMVs (**Figure 3**).^112^, ^113^ In contrast, sphingolipid (SL), another lipid in* P. gingivalis* OMVs, plays a different role than the virulence factors described above. SL in *P. gingivalis* OMVs inhibits the immune response and facilitates immune escape.^114^ SL-depleted *P. gingivalis* OMVs significantly increase the expression of TNF-α, IL-1β, and IL-10 in THP-1 cells, confirming that SL in *P. gingivalis* OMVs partially inhibits the host immune response.^114^ SL may facilitate the immune escape of *P. gingivalis* by altering the cargo of OMVs and affecting the important receptors Toll-like receptor (TLR)2 and TLR4 in the periodontium and their signalling pathways (**Figure 3**).^115^-^121^

*P. gingivalis* OMVs can also interact with other oral bacteria to help parental bacteria function synergistically with other oral pathogens in adhesion, spread, and serum survival. The adhesion of *T. forsythia* to epithelial cells relies on its cell surface-associated protein BspA, and *P. gingivalis* OMVs facilitate this attachment.^56^ *P. gingivalis* OMVs also promote the adhesion and diffusion of the spirochetes *T. denticola* and *Lachnoanaerobaculum saburreum.*^122^ In human immortalized oral epithelial cells,* P. gingivalis* OMVs prevent *F. nucleatum* from entering oral epithelial cells and being degraded intercellularly.^123^ In turn, *P. gingivalis* invasion is accelerated by this process to promote deep-seated bacterial infection.^124^ These different effects may result from the attempt to maximize the efficiency of promoting inflammation in different host cells and in different intracellular environments. Pretreating *C. ochracea* incubated with human serum with *P. gingivalis* OMVs revealed that *P. gingivalis* OMVs inhibited the bactericidal activity of the serum against *C. ochracea.*^125^ This ability of *P. gingivalis* OMVs might be broad enough to protect not only *C. ochracea* but also other oral pathogenic bacteria that participate in the synergistic pathogenic process of periodontitis.^125^

### 3.2 *T. forsythia* OMVs

*T. forsythia* is a gram-negative oral pathogen associated with advanced periodontitis 126 and a member of the “red complex”.^127^ *T. forsythia* OMVs carry a variety of virulence factors through the T9SS and glycosylation.^112^, ^128^ Like *P. gingivalis*, *T. forsythia* possesses a T9SS that transfers cargo proteins into OMVs.^98^, ^129^-^131^ OMVs also depend on the glycosylation of virulence-associated proteins to cause disease.^132^ *T. forsythia* has an O-type glycosylation system, and locus analyses indicate that glycosylation occurs on a vast range of motifs, including (D) (S/T) (A/I/L/V/M/T/S/C/G/F).^133^ Thus, there is a high probability of potential glycoproteins in bacteria and their OMVs.^133^ The major glycoprotein class observed in *T. forsythia* OMVs is the cargo proteins of the T9SS.^133^

*T. forsythia* OMVs can play a regulatory role in the immune processes of dendritic cell activation and CD4^+^ T-cell differentiation. Unlike *P. gingivalis* OMVs, *T. forsythia* OMVs promote the secretion of proinflammatory cytokines by BMDCs and induce Th1 differentiation in naïve CD4^+^ T cells.^93^

### 3.3 *T. denticola* OMVs

Treponema is an important factor in different human chronic diseases, and *T. denticola* is a common motile, specialized anaerobic gram-negative treponema that is a member of the “red complex” and a major periodontitis pathogen.^127^, ^134^, ^135^ EVs are often found around *T. denticola*, in which proteins and protein hydrolysis patterns are quite similar to those of the bacterial sheaths.^136^ Thus, *T. denticola* OMVs may originate from the sheath of bacteria through flagellar rotation.^136^ The biogenesis of *T. denticola* OMVs might be triggered by limited exposure to the major sheath protein (Msp).^21^, ^137^ Since Msp forms an array within the bacterial OM and is mostly periplasmic with limited surface exposure, the generation of OMVs may be a strategy to increase Msp surface exposure.^137^

OMVs of *T. denticola* act as long-range virulence factors and contain the necessary adhesins and proteolytic arsenal for adhering to and damaging host cells and affecting inflammation.^136^ The major virulence factors in *T. denticola* OMVs include dentilisin, Msp, and lipooligosaccharide (LOS).^68^, ^138^ Dentilisin is located on the surface of *T. denticola* OMVs. It can degrade tight junction proteins such as ZO-1, disrupt transepithelial resistance (TER), and effectively enter human epithelial type 2 (HEp-2) cells.^68^, ^138^ Msp in *T. denticola* OMVs enhances the inhibition of neutrophils to perpetuate periodontal inflammation.^139^ It moderately reduces Src homology 2 domain-containing inositol phosphatase 1 (SHIP1) activity and prevents the secondary activation of the phosphatase and tensin homologue (PTEN)/phosphatidylinositol 3-kinase (PI3K) response, which restricts phosphatidylinositol phosphate (PIP) signalling to disrupt the phagocytic function of neutrophils and evade host immune responses.^139^ Like *P. gingivalis*, *T. denticola* OMVs activate BMDCs and indirectly induce Th17 differentiation of naïve CD4^+^ T cells.^93^ However, there is no significant alteration in the levels of proinflammatory factors during the activation of BMDCs, which may be related to the highly proteolytic characteristics of *T. denticola* OMVs and their subsequent posttranslational degradation.^93^

### 3.4 *A. actinomycetemcomitans* OMVs

*Aggregatibacter actinomycetemcomitans* is an inactive, nonencapsulated, slow-growing gram-negative anaerobic bacterium.^140^ It is capable of producing OMVs that possess a bimodal size distribution.^141^ The composition of these OMVs is complex, and includes virulence factors, tentative virulence-associated proteins, and small molecules.^142^ Biologically active virulence proteins of *A. actinomycetemcomitans* OMVs include the unique exotoxins CDT and LtxA.^143^-^145^ Junction-related proteins on *A. actinomycetemcomitans* OMVs are fused to lipid rafts on the host plasma membrane, and the OMVs are then internalized, delivering virulence factors such as CDT to susceptible host cells in the periodontium.^26^ CDT subsequently enters the nucleus and cleaves double-stranded DNA, leading to rapid growth arrest and thus destruction of periodontal tissues.^26^ LtxA is thought to be the major virulence molecule in invasive periodontitis caused by *A. actinomycetemcomitans*^146^-^148^, and it is involved in the formation of* A. actinomycetemcomitans* OMVs and is enriched in OMVs.^25^, ^149^ This enrichment may partly explain the size dependence of toxin distribution in *A. actinomycetemcomitans* OMVs, with larger OMVs being more likely to contain LtxA.^141^ By killing defence cells, LtxA in OMVs shields bacteria from phagocytosis, thereby strengthening the protective effect.^150^ OMVs also contain peptidoglycans that can activate the nucleotide-binding oligomerization domain (NOD)1 and NOD2.^151^ When internalized by nonphagocytic cells, OMVs act as promoters of innate immunity.^151^ Mass spectrometry analysis has revealed a series of potential virulence-related proteins in *A. actinomycetemcomitans* OMVs, including BilRI, Omp100, TdeA, and ferritin-like proteins.^152^, ^153^ In addition, small molecules in* A. actinomycetemcomitans* OMVs are important for pathogenesis. LPS and lipid-associated proteins can promote bone resorption.^154^, ^155^ msRNAs capable of signal transduction can enhance the activation of NF-κB via TLR-8 and promote TNF-α production in macrophages to strengthen the inflammatory response.^33^, ^156^

*A. actinomycetemcomitans* OMVs protect their parental bacteria as well as other bacteria. Serum resistance, which enables bacteria to escape from the innate immune system, is crucial for bacteria to enter the blood and cause infection.^157^-^160^ *A. actinomycetemcomitans* OMVs serve as immune targets that strongly activate and deplete complement via virulence factors such as LPS.^161^ In this way, serum resistance is induced, and *A. actinomycetemcomitans* is protected.^161^ *A. actinomycetemcomitans* OMVs may also protect against other pathogens.^125^ Experiments could be designed to test the protective efficacy of *A. actinomycetemcomitans* OMVs against other periodontal pathogens in the serum.

### 3.5 *F. nucleatum* OMVs

*F. nucleatum* is a gram-negative, anaerobic, adhesive bacterium commonly found in the oral mucosa and is involved in biofilm formation.^162^, ^163^ *F. nucleatum* OMVs can regulate the extent of the inflammatory response and tissue barrier permeability.^164^ Experiments in which* F. nucleatum* OMVs stimulated bone marrow-derived macrophages (BMDMs) revealed that *F. nucleatum* OMVs could upregulate TNF-α and iNOS to induce significant proinflammatory features and oxidative stress in macrophages, promoting the polarization of M0 macrophages towards proinflammatory M1 macrophages. In this way, *F. nucleatum* OMVs accelerate the formation of an inflammatory microenvironment. When mouse gingival fibroblasts (MGFs) were isolated and cocultured with *F. nucleatum* OMV-treated macrophages, *F. nucleatum* OMV-treated macrophages caused more significant MGF damage than did macrophages cocultured directly with *F. nucleatum* OMVs, suggesting that the inflammatory microenvironment enhances the damaging effect of *F. nucleatum* OMVs on MGFs. Researchers further conducted *in vivo* experiments with periodontitis mouse models and reported that *F. nucleatum* OMV-treated periodontitis model mice presented greater numbers of osteoclasts, more widespread alveolar bone damage, and significant increases in the levels of IL-1β, IL-6, and TNF-α. These changes demonstrated the role of *F. nucleatum* OMVs in exacerbating periodontal inflammation, which may be achieved by shifting M0 macrophages to the M1 phenotype to promote the inflammatory microenvironment.^165^ In addition, *F. nucleatum* OMVs can disrupt the oral mucosal epithelial barrier and further enhance the spread of virulence by downregulating the tight junction protein claudin-4 in HOKs.^166^ A recent study in our lab revealed that *F. nucleatum* OMVs could directly induce the deterioration of periodontitis by enhancing inflammation of the periodontium and the absorption of alveolar bone, which was almost equivalent to the effect of *F. nucleatum* itself.^13^ As an entity of multiple pathogenic components, *F. nucleatum* OMVs interact with human periodontal ligament stem cells (HPDLSCs) and trigger the NLRP3 inflammasome, thus reducing the accumulation of mineralization in HPDLSCs and promoting the resorption of alveolar bone.

### 3.6 *C. ochracea* OMVs

*C. ochracea* is a facultative anaerobic gram-negative bacillus responsible for the early stages of plaque formation and is opportunistically pathogenic for periodontal infections.^167^ The biogenesis of *C. ochracea* OMVs is associated with the enrichment of the unsaturated fatty acid phosphatidylinositol (PI) and increased membrane fluidity.^168^ Recent research has shown that *C. ochracea* can undergo a unique microbial extracellular electron transfer (EET) process through the OM.^169^ *C. ochracea* OMVs, which originate from and share high similarity with the OM, might also be capable of mediating EET and affecting the metabolism of other oral bacteria through long-distance electron transfer. Thus, the isolation of *C. ochracea* OMVs for study is needed to confirm this process. To date, little is known about *C. ochracea* OMVs, and only their biogenesis and potential roles have been investigated. Future work should shed light on the virulence factors, functions, and mechanisms involved.

In summary, *P. gingivalis* is the keystone pathogen of periodontitis, and studies on its OMVs are more extensive than those on other oral pathogens. The virulence factors in *P. gingivalis* OMVs include proteases, nucleic acids, and lipids, and their functions can be divided into two main categories: structural destruction and effects on the host immune response. Structural destruction, such as the apoptosis of HPDLCs caused by gingipains in *P. gingivalis* OMVs, can promote the spread of OMVs via a positive feedback mechanism. The proinflammatory effects of OMVs, which regulate the host immune response, are more extensive. *P. gingivalis* OMVs promote the expression of proinflammatory factors in nonimmune cells such as gingival epithelial cells. They also interact with PRRs to activate nonspecific immunity. In addition, *P. gingivalis* OMVs activate BMDCs and indirectly induce Th17 polarization of naïve CD4^+^ T cells to affect specific immunity. Although the general role of *P. gingivalis* OMVs is to exacerbate inflammation, measures are available to protect parental bacteria from immune attack, even when the overall immune response is strengthened. *P. gingivalis* OMVs can moderately inhibit the SL-induced immune response, as a strategy to achieve immune escape. Prestimulation of monocytes with *P. gingivalis* OMVs elicits immune tolerance. When these monocytes are exposed to *P. gingivalis*, immune escape, rather than an immune response, occurs. *P. gingivalis* OMVs can also help bacteria escape death by degrading and depleting bactericidal substances such as complement, MPO, and LL-37.

Although the role of *P. gingivalis* OMVs has been widely studied in both structural and immunologic dimensions, the complicated underlying mechanisms still need to be fully elucidated. Some studies have explored the effects of *P. gingivalis* OMVs as a whole, but the specific molecules involved are unclear. Further analysis is needed. For example, we can first apply heat treatment to determine the thermal stability of unknown molecules and then further analyse the thermal stability with the control variable method to verify the results. If the OMV-mediated effect disappears after heat treatment, a molecule, such as a protein, is heat unstable. If there is no significant change, a molecule, such as LPS, is heat stable. If there is a partial change, it may be a synergistic effect of both heat-unstable molecules and heat-stable molecules.

In addition to *P. gingivalis*, the OMVs of other oral pathogens, including *T. forsythia*, *T. denticola*, *A. actinomycetemcomitans*, *F. nucleatum*, and *C. ochracea,* also play a pathogenic role. Similar to *P. gingivalis* OMVs, the OMVs of these bacteria exert their effects mainly through structural destruction and inflammation and can induce immune escape to protect against pathogens. Structurally, dentilisin in *T. denticola* OMVs degrades the tight junction protein ZO-1 to disrupt intercellular tight junctions. In terms of inflammation, OMVs from these pathogenic bacteria function similarly to *P. gingivalis* OMVs do, enhancing the host immune response by inducing the overexpression of proinflammatory factors. These OMVs also possess immune escape mechanisms. *A. actinomycetemcomitan*s OMVs kill defence cells to protect bacteria via LtxA. They also act as complement targets to induce serum resistance and prevent bacteria from being attacked. However, only a few studies have focused on oral OMVs other than *P. gingivalis* OMVs, and there is a dearth of information on their interrelationships and systemic effects. In-depth studies on these OMVs are needed.

## 4. Pathogenic roles of oral bacterial OMVs in diseases

Oral biofilms, also known as dental plaques, are complex biofilms arranged in spatial order on the tooth surface and gum by bacteria.^170^ Usually, the oral biofilm is dynamically balanced, with constant interactions between the oral microbiome and the host.^171^ Disruptions in the balance triggered by pathogens can lead to many oral diseases, including dental caries 172, periodontitis 173-175, mucosal diseases 176, and oral cancer 177, 178, as well as other health issues, such as circulatory diseases and endocrine diseases.^179^

In recent years, intensive research interest has focused not only on the role of pathogenic gram-negative bacteria in oral biofilms but also on their secreted OMVs. OMVs of oral pathogens play an essential role in periodontitis and OSCC, facilitating their progression and systemic spread. However, the specific functions and mechanisms still warrant further investigation. By summarizing the pathogenic roles of OMVs from major oral pathogens and discussing their therapeutic potential, we attempt to provide possibilities for exploring the pathogenesis, systemic spread, and biological therapies of periodontitis and OSCC.

### 4.1 OMVs in periodontitis

Periodontitis is a form of chronic inflammation related to biofilm dysregulation.^180^, ^181^ It is the result of imbalanced periodontal ecology, in which pathogens cause destruction either directly by invading periodontal tissue or indirectly by affecting the immune system.^181^ Periodontitis is characterized by the gradual destruction of dental supporting structures, such as the alveolar bone, periodontal ligament, and cementum. In cases of severe deterioration, periodontitis eventually leads to tooth loss.^175^, ^180^

Periodontitis is initiated by interactions among multiple pathogenic bacteria.^182^ The oral pathogenic bacteria *P. gingivalis*, *T. forsythia*, and *T. denticola* constitute the "red complex" consortium.^127^ They congregate, interact, and metabolically intertwine with each other, forming a dense biofilm to trigger and aggravate periodontitis.^127^, ^132^, ^183^ The key pathogens involved in periodontitis also include *A. actinomycetemcomitans*, *F. nucleatum*, and *C. ochracea.*^167^, ^184^, ^185^ These gram-negative periodontal bacteria not only attack the periodontium but also produce OMVs to accelerate periodontitis progression. OMVs inherit many characteristics from their parental bacteria and even perform better in different ways. These OMVs transfer virulence factors into host cells to promote periodontitis (**Table 1**). They also interact with other bacteria, causing changes in microbial communities and consolidating the dominant position of pathogenic bacteria. OMVs even associate periodontitis with other nonoral diseases by promoting the occurrence of systemic diseases via their strong ability to spread.

Some *in vitro* experiments have revealed that OMVs from *P. gingivalis* can inhibit HGF proliferation and affect inflammation in human gingival epithelial cells (HGECs) (**Figure 3**).^73^, ^186^ Further evidence has been derived from animal model studies. Compared with that in untreated rats, significant resorption of alveolar bone was detected, suggesting that *P. gingivalis* OMVs promote periodontitis.^108^ *P. gingivalis* OMVs also work together with other periodontal pathogens to aggravate periodontitis.^125^

The bacterial virulence factors in OMVs play a key role in periodontal inflammation and tissue destruction caused by OMVs. Gingipains in *P. gingivalis* OMVs can trigger the detachment of oral squamous epithelial cells to promote periodontitis.^24^ As OMVs diffuse away from the periodontal pocket, the environmental oxygen level increases, and gingipains are inactivated because of the oxidation of cysteine residues.^187^, ^188^ However, gingipains that lose protein hydrolysis activity can still damage periodontal tissues via a mechanism different from that in the periodontal pocket.^189^, ^190^ Inactive RgpA interacts with the epidermal growth factor receptor (EGFR) on the membrane surface of human telomerase-immortalized gingival keratinocyte (TIGK) cells and phosphorylates tyrosine residue Y1173 of the receptor.^189^ It causes a robust proinflammatory response with a marked increase in TNF-α and IL-1β and activates the PI3K and protein kinase B (AKT) pathways.^189^, ^191^, ^192^ In the dendritic cells of periodontal tissues, the inactive RgpA-stimulated AKT pathway strongly triggers local inflammation around the alveolar bone, causing destruction and resorption.^189^ Moreover, via this AKT pathway, inactive RgpA may regulate apoptosis. In the intracellular pathogen *Shigella flexneri*, IpgD-induced phosphorylation of AKT inhibits apoptosis and promotes intracellular survival and growth.^193^ Since *P. gingivalis* is also an intracellular pathogen, it is hypothesized that inactive RgpA in *P. gingivalis* OMVs may also promote intracellular survival through the AKT pathway (**Figure 3**).^194^, ^195^ LPS in *P. gingivalis* OMVs interacts with PRRs on fibroblasts, epithelial cells, and macrophages in periodontal tissues.^110^, ^111^ This interaction functions in the chronic inflammatory pathology of periodontitis by activating inflammasome complexes to destroy connective tissue and cause alveolar bone resorption.^196^ Fibrinolytic activity is critical for human periodontal health.^197^-^199^ *T. forsythia* OMVs can release a significant virulence protein named miropin**,** which can inhibit human fibrinolysin and prevent fibrin clot degradation.^200^ This may have a deleterious effect on periodontal tissue. LOS resides on the surface of *T. denticola* OMVs and has a high affinity for laminin.^201^ It assists *T. denticola* OMVs in adhering to gingival epithelial cells and fibroblasts, and damages periodontal tissues.^201^ LOS can also help *T. denticola* OMVs bind to other bacteria, increasing their proinflammatory potential. Applying *T. denticola* LOS-coated *Peptostreptococcus micros* to stimulate HGFs can significantly increase the expression of IL-6 and IL-8.^201^ msRNAs in *T. denticola* OMVs can act as novel signalling molecules that mediate signal transmission to bacteria and host cells.^33^ However, its specific functions and mechanisms have not yet been elucidated.

Progress in the field of OMVs has provided increasing detail about the pathogenic role of oral bacterial OMVs in periodontitis. Several lines of evidence have suggested that OMVs derived from periodontal pathogens such as *P. gingivalis* and *T. denticola* participate in periodontal tissue destruction. However, the differences in OMV components and comparisons of the OMV virulence of different oral bacteria are still lacking. The precise process and key molecular events involved in OMV-induced periodontitis still need to be elucidated.

### 4.2 OMVs in OSCC

OSCC is one of the most common malignancies.^202^ It occurs mainly in the tongue, mouth floor, gingiva, and buccal mucosa, with various subtypes and high rates of recurrence and metastasis.^203^ Chronic infection is a significant risk factor for cancer development, and host-microbiota interactions in cancer are closely related to tumorigenesis, progression, metastasis, therapeutic efficacy, and prognosis.^204^, ^205^ Therefore, the pathogenesis of OSCC is associated with the dysregulation of oral bacterial ecology and inflammation. Studies have shown that OSCC strongly correlates with pathogenic bacteria, including *P. gingivalis*, *F. nucleatum*, and *T. denticola*.^206^-^209^ The progression of OSCC is accelerated by promoting epithelial cell proliferation 210-212, enhancing cancer cell invasion and metastasis 213, 214, and modulating the immune microenvironment.^215^, ^216^ OMVs have also been shown to be involved in OSCC progression in recent studies (**Table 1**).

**Figure 4:**
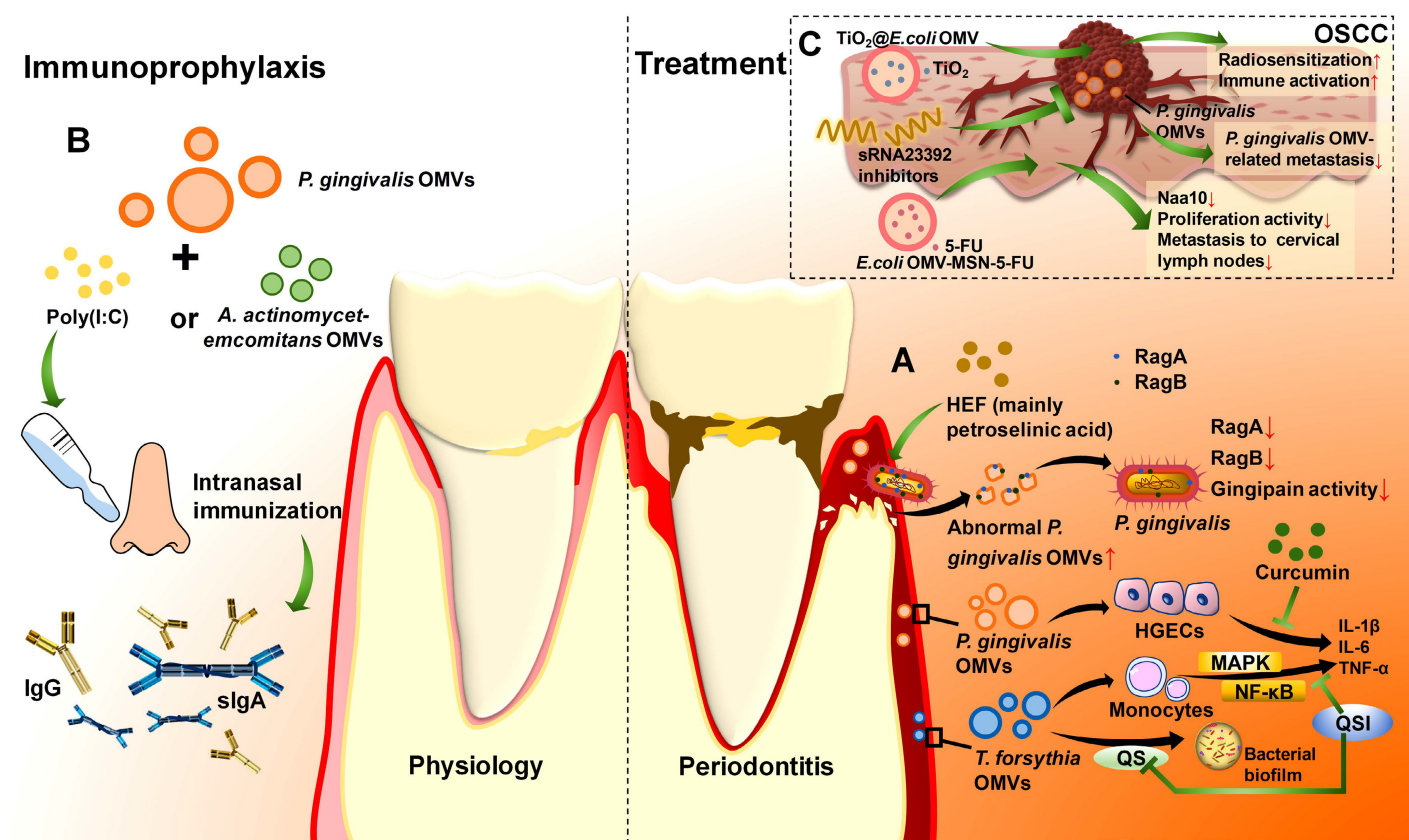
** The role of OMVs in the initiation, progression, and metastasis of OSCC.** (**A**). *P. gingivalis* OMVs can enter oral squamous cell carcinoma cells and release sRNA23392, which then binds to DSC2 mRNA for degradation, leading to a reduction in the transmembrane adhesion molecule DSC2. Intercellular adhesion decreases, and the invasiveness of OSCC cells increases. (**B**). *F. nucleatum* OMVs can interact with Claudin-1 in the nucleus, which upregulates N-cadherin and vimentin and downregulates E-cadherin, subsequently causing epithelial-mesenchymal transition in OSCC cells to promote pulmonary metastasis.

*P. gingivalis* OMVs disrupt intercellular adhesion to increase cell invasiveness and metastasis in epithelial malignancies.^217^, ^218^ Desmosomes are essential intercellular adhesive complexes in epithelial and some nonepithelial tissues, and serve as reliable tumour markers.^219^ Desmocollin 2 (DSC2) is a cadherin-type transmembrane adhesion molecule whose aberrant expression is correlated with the invasive properties of cancers 220, and its mRNA can be bound to and degraded by the virulence factor sRNA23392 in *P. gingivalis* OMVs.^217^ This process reduces the expression of DSC2, decreases the adhesion among oral carcinoma HSC-3 cells, and leads to an increase in migration and invasion, ultimately resulting in the metastasis of OSCC (**Figure 4A**).^217^

*F. nucleatum* OMVs may trigger the lung metastasis of OSCC by inducing the epithelial-mesenchymal transition (EMT) phenotype in cancer cells.^12^ EMT is closely related to cancer metastasis.^221^, ^222^ The epithelial marker E-cadherin and the tight junction protein ZO-1 are downregulated in cancer, whereas the mesenchymal markers N-cadherin and vimentin are upregulated.^221^ In the OSCC hormonal mouse model, metastatic lung nodules were present on the lung surface in the *F. nucleatum* OMV-treated group but did not appear in the control group, suggesting that *F. nucleatum* OMVs can promote the lung metastasis of OSCC (**Figure 4B**).^12^ A report involving coculture of the human tongue squamous carcinoma cell line CAL27 and the oral carcinoma cell line HSC-3 with *F. nucleatum* OMVs indicated that *F. nucleatum* OMV exposure could downregulate E-calmodulin expression and upregulate vimentin expression in these two cell lines, suggesting that *F. nucleatum* OMVs induce an EMT phenotype in OSCC cells to accelerate their lung metastasis. This process is achieved by activating autophagic pathways to increase autophagic flux; thus, the *F. nucleatum* OMV-induced EMT phenotype and metastasis of OSCC cells can be reversed by using autophagy inhibitors such as chloroquine (CHQ).^12^

### 4.3 Systemic effects of OMVs

**Figure 5:**
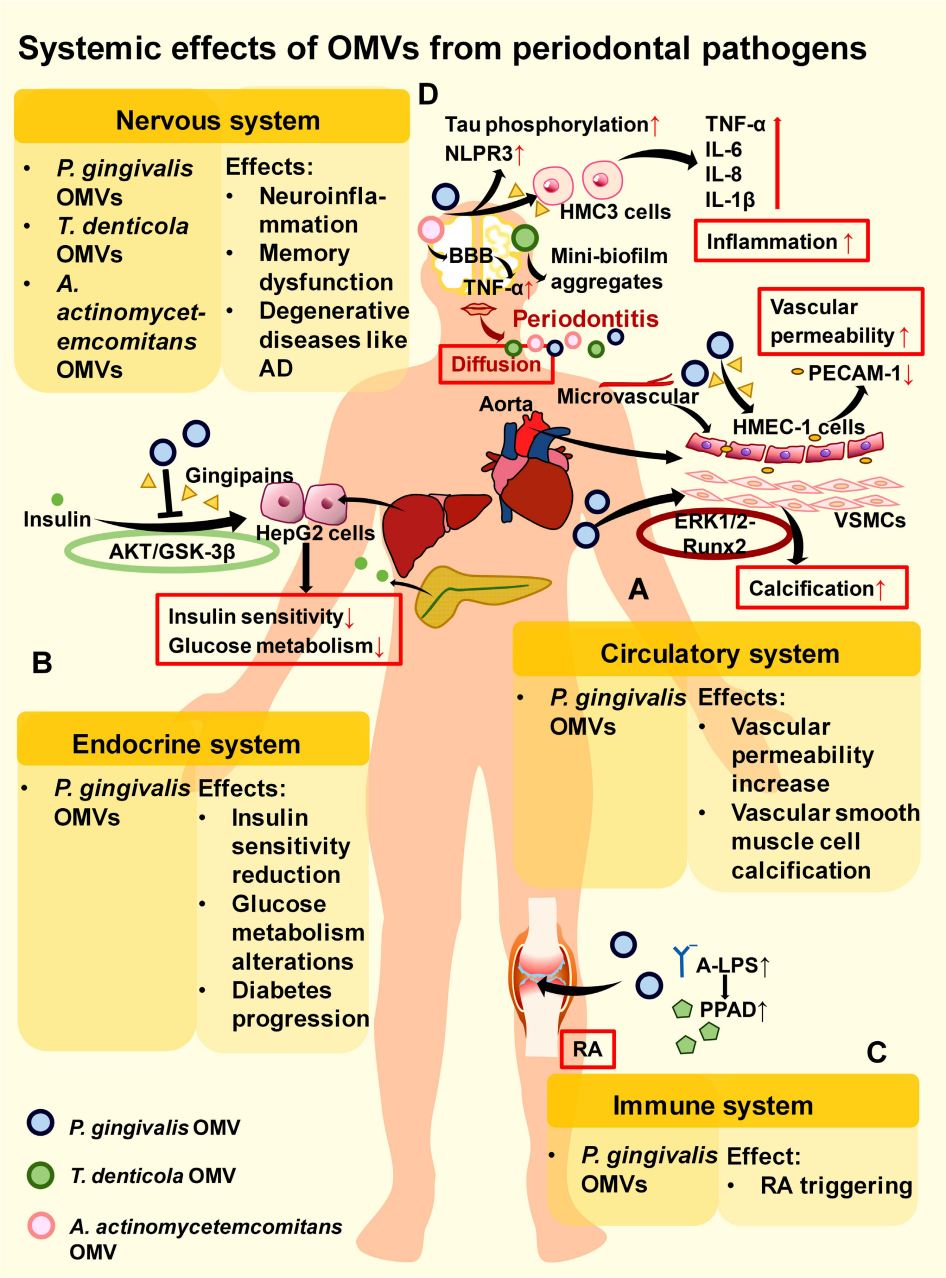
** Systemic effects of OMVs derived from oral bacteria. OMVs from oral bacteria can diffuse and affect the nervous system, circulatory system, endocrine system, and immune system.** (**A**). In the circulatory system, gingipains in *P. gingivalis* OMVs can reduce PECAM-1 on the surface of HMEC-1 cells and increase vascular permeability. *P. gingivalis* OMVs also promote the calcification of VSMCs through the ERK1/2-Runx2 pathway. (**B**). In the endocrine system, *P. gingivalis* OMVs inhibit the AKT/GSK-3β pathway and reduce the insulin sensitivity of HepG2 cells through gingipains, leading to alterations in glucose metabolism in the liver. (**C**). In the immune system, A-LPS on the surface of *P. gingivalis* OMVs may participate in PPAD sorting, and increased PPAD is potentially associated with the occurrence of RA. (**D**). In the nervous system, *P. gingivalis* OMVs can lead to neuroinflammation by promoting the expression of proinflammatory factors in HMC3 cells, Tau phosphorylation, and NLRP3 inflammasome production. OMVs from *T. denticola* may help form mini-biofilm aggregates. *A. actinomycetemcomitans* OMVs may cross the BBB and promote the expression of the proinflammatory factor TNF-α.

Periodontitis is linked through *P. gingivalis* OMVs to a variety of systemic impairments, including circulatory diseases, endocrine diseases, autoimmune diseases, and neurologic diseases.^30^, ^77^, ^223^ In circulatory diseases, *P. gingivalis* OMVs can induce oedema and vascular calcification. They decrease the level of platelet endothelial cell adhesion molecule 1 (PECAM-1) on the surface of human microvascular endothelial cells (HMEC-1) via gingipains.^224^ PECAM-1 acts as an endothelial adhesion molecule crucial for sustaining vascular integrity at cell-cell junctions.^225^ Thus, its loss causes increased vascular permeability and leakage, leading to cellular oedema (**Figure 5A**).^224^ Moreover, this damage to cellular junctions caused by *P. gingivalis* OMVs may expose connective tissues and contribute to platelet activation, subsequently activating immune cells in the endothelium and increasing the risk of systemic diseases.^226^ *P. gingivalis* OMVs also induce calcification of vascular smooth muscle cells (VSMCs) through the ERK1/2-Runx2 pathway in the mouse aorta.^75^ Notably, the nanoscale size of *P. gingivalis* OMVs may even allow vascular injury to occur in tiny spaces inaccessible to bacteria (**Figure 5A**).^224^ In endocrine diseases, after translocating to the liver, *P. gingivalis* OMVs, with the help of gingipains, downregulate the insulin-induced AKT/glycogen synthase kinase-3 β (GSK-3β) pathway in HepG2 cells, attenuate insulin sensitivity, and alter glucose metabolism, which ultimately leads to diabetes progression (**Figure 5B**).^36^, ^227^, ^228^

*P. gingivalis* OMVs have also been potentially implicated in autoimmune diseases. Since *P. gingivalis* is closely connected to the pathogenesis of RA and one probable cause of RA is the loss of tolerance to citrullinated proteins, *P. gingivalis* OMVs might function to extend periodontitis to RA through the key enzyme PPAD.^229^ This process may rely on A-LPS modification of the bacterial OM to affect PPAD anchoring and distribution to OMVs, thereby indirectly influencing RA initiation (**Figure 5C**).^77^ In the nervous system, *P. gingivalis* OMVs increase the secretion of proinflammatory cytokines by human microglial clone 3 (HMC3) cells and further induce neuroinflammation via neurotoxic gingipains (**Figure 5D**).^76^ After oral gavage, *P. gingivalis* OMVs enter the ventricles of mice to promote inflammation and tau phosphorylation in the brain (**Figure 5D**).^76^ They may also induce trigeminal nerve-mediated cognitive impairment.^230^ These results indicate that *P. gingivalis* OMVs can act as vital mediators of periodontitis to affect the nervous system and trigger degenerative diseases such as AD.

However, the lack of *in vivo* experiments prevents further verification of these systemic effects induced by *P. gingivalis* OMVs. Controlled experiments can be designed in mice to establish the systemic spread process. For example, to verify whether A-LPS modification can affect the anchoring of PPAD to OMVs and indirectly promote RA initiation, we can divide *P. gingivalis* into A-LPS-modified and non-A-LPS-modified groups, inject them separately into mice, extract OMVs, calculate their PPAD content, and observe the occurrence of RA. Additionally, more clinical data are needed to clarify the functions of these genes in the host body.

*P. gingivalis* OMVs can also interact with other pathogens and cause systemic effects. For example, with the aid of *P. gingivalis* OMVs, HIV-1 can rapidly spread to HOKs and promote oral mucosal infection. This faster rate of *P. gingivalis* OMV-mediated viral entry, reverse transcription, and integration may be related to the more rapid entry of OMVs into host cells than that of the virus alone.^42^ *P. gingivalis* OMVs induce reversible *Staphylococcus aureus* aggregation and promote its adherence to neutrophils with subsequent internalization, which indirectly allows *S. aureus* to be transported into the bloodstream and potentially triggers *S. aureus* bacteraemia (SAB)^.41, 231, 232^ However, as mentioned above, *P. gingivalis* OMVs enhance the proliferative activity of *F. nucleatum* and protect *C. ochracea* from serum bactericidal activity rather than promoting its internalization. This difference may be determined by the pathogenicity of the bacteria in periodontitis. *P. gingivalis* OMVs have synergistic effects with their copathogenic periodontal pathogens. For nonperiodontitis pathogens such as *S. aureus*, *P. gingivalis* OMVs may promote their internalization by neutrophils to eliminate their competition in the periodontium, thus indirectly triggering systemic diseases such as SAB. However, this possibility requires further investigation via controlled experiments involving* P. gingivalis* OMVs against different oral pathogens (especially periodontal pathogens) and nonoral pathogens to verify whether there are distinct differences.

*T. denticola* has been shown to be an essential mediator of the spread of periodontal infection to the nervous system, leading to neurodegeneration in the midbrain. Treponemes are neurotrophic: they can reach the brain from a peripheral route of infection. Interactions between virulence factors of treponemes and microglia can directly or indirectly inflict neurological damage.^233^ *T. denticola* has been reported to form mini-biofilm aggregates after entering the CNS, increasing its virulence through group sensing.^233^ Since *T. denticola* OMVs spread faster and can promote biofilm formation than can the whole bacteria, *T. denticola* OMVs possibly participate in mini-biofilm formation and neurological damage in the CNS (**Figure 5D**).

*A. actinomycetemcomitans* OMVs also influence the development of neurological disorders. Research on mice has shown that periodontopathogenic *A. actinomycetemcomitans* OMVs not only promote TNF-α secretion by human macrophages in the periodontium but also pass through the blood‒brain barrier (BBB) to increase TNF-α levels in the brain, contributing to the progression of neuroinflammatory disorders such as AD (**Figure 5D**).^156^

## 5. Therapeutic potentials of OMVs

OMVs of periodontal pathogens are rich in various virulence factors that are closely connected with parental bacteria and other pathogenic bacteria. These pathogenic effects strongly suggest the great therapeutic potential of OMVs as drug targets and as high-antigenicity vaccines for periodontitis treatment, and eliminating OMVs is beneficial for preventing the aggravation and spread of periodontitis and OSCC (**Table 2**).

### 5.1 Therapeutic potential of OMVs in periodontitis

**Figure 6:**
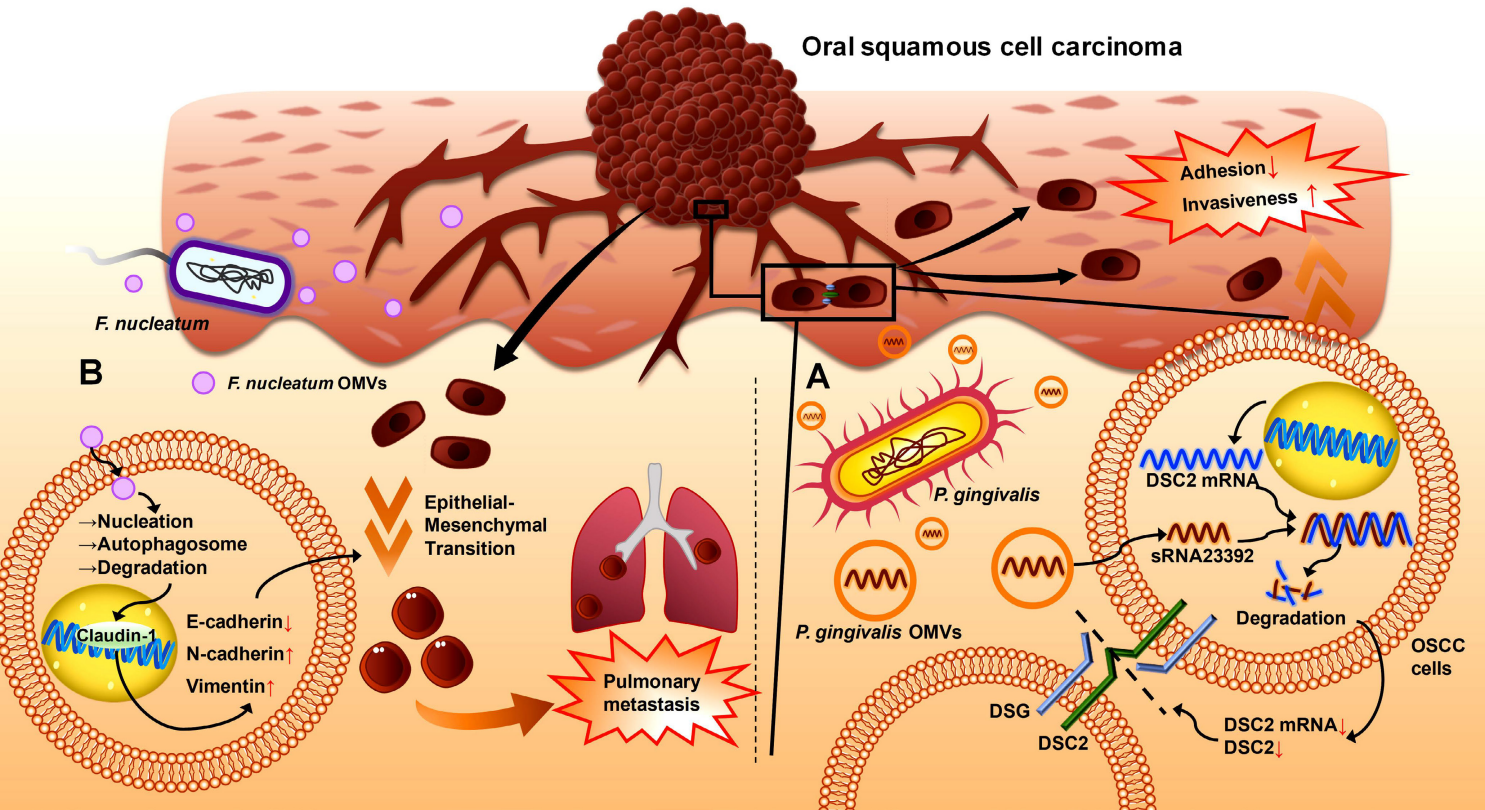
** Therapeutic potential of OMVs in periodontitis and OSCC.** (**A**). Drugs can target OMVs for the treatment of periodontitis. HEF promotes the production of excess abnormal OMVs by *P. gingivalis*, which in turn downregulates the expression of the important OM transport proteins RagA and RagB in bacteria, decreases gingipain activity, and ultimately disrupts nutrient cycling. Curcumin can suppress the expression of the proinflammatory cytokines IL-6, IL-1β, and TNF-α in HGECs induced by *P. gingivalis* OMVs. QSIs can block QS, which may function partly through *T. forsythia* OMVs, to inhibit bacterial biofilm formation. It also blocks the MAPK and NF-κB signalling pathways to suppress the proinflammatory cytokine production induced by *T. forsythia* OMVs in monocytes. (**B**). *P. gingivalis* OMVs can be used as antigens in periodontitis vaccines. With immune adjuvants such as poly(I:C) and the more efficient *A. actinomycetemcomitans* OMVs, IgG in the serum and s-IgA in the mucosa can be induced via intranasal immunization. (**C**). *E. coli* OMVs can act as carriers for 5-FU and TiO_2_ in OSCC. The system *E. coli* OMV-MSN-5-FU effectively decreases the expression of the cancer progression biomarker Naa10 and inhibits the proliferation and metastasis of cancer cells. The system TiO2@*E.coli* OMV enhances the radiosensitization and immune activation of the tumour tissue.* P. gingivalis* OMVs can act as a target of sRNA23392 inhibitors to inhibit the *P. gingivalis* OMV-related metastasis.

*P. gingivalis* OMVs are potential therapeutic targets for periodontitis. HEF is rapidly lethal to* P. gingivalis* and displays significant antibacterial activity.^78^ Low concentrations of HEF lead to the formation of OMV-like bubbles, whereas high concentrations promote bacterial disintegration, resulting in the overproduction of OMVs with aberrant surface properties.^78^ RagA and RagB, two transport proteins of *P. gingivalis* and its OMVs, are essential for nutrient acquisition. Excess OMV production induced by HEF results in the overrelease of RagA and RagB from bacteria into OMVs and the depletion of the vital RagA/RagB conveyance mechanism in *P. gingivalis*, and eventually, the bacteria become undernourished.^78^ HEF has also been found to have inhibitory effects on both Rgps and Kgp in *P. gingivalis* OMVs, which hinders periodontitis progression (**Figure 6A**).^78^ Another drug, curcumin, can inhibit the proinflammatory and toxic effects of *P. gingivalis* OMVs.^79^ A report revealed that curcumin significantly suppressed *P. gingivalis* OMV-mediated secretion of IL-6, IL-1β, and TNF-α by HGECs in a dose-dependent manner.^79^ Moreover, curcumin inhibits the ability of OMVs to adhere to and enter HGECs and restrains their toxic effects on cell migration, preventing the death or apoptosis of host cells and effectively restraining periodontitis progression (**Figure 6A**).^79^

*T. forsythia* OMVs can also serve as biotherapeutic targets. Quorum sensing (QS), a form of cell density-dependent communication among bacteria, promotes a wide range of bacterial activities, such as biofilm formation and virulence expression, which provides an advantage for bacterial survival.^234^ Quorum-sensing inhibitors (QSIs) are able to block QS and prohibit biofilm formation. Using d-arabinose and d-galactose as community QSIs to treat *T. forsythia* OMVs reduces the NF-κB and MAPK pathways, resulting in decreased TNF-α, IL-1β, IL-6, and IL-8 production by THP-1 monocytes.^235^ This result suggests that *T. forsythia* OMVs may partly mediate QS and that QSIs not only inhibit dental plaque formation but also restrain the proinflammatory response stimulated by *T. forsythia* OMVs (**Figure 6A**).^235^ QSIs may also have excellent potential to function on OMVs of other periodontal pathogens, showing great promise in periodontitis biotherapy. For example, as the process by which *T. denticola* diffuses into the CNS to form a mini-biofilm by QS may be associated with *T. denticola* OMVs, developing *T. denticola* OMV-associated QSIs to restrain QS in the CNS can clarify the role of *T. denticola* OMVs in CNS mini-biofilm formation and alleviate neurological injuries caused by periodontitis.^233^

Furthermore, in addition to eliminating OMVs in biotherapy, processing modified OMVs is a novel method for periodontitis vaccine development since OMVs possess high antigenic properties to stimulate the host immune response. OMVs migrate across the extracellular matrix to the lymph nodes and are then taken up by dendritic cells.^236^-^239^ Their hardness contributes to the slow release of antigens, eliciting a long-lasting immune response.^240^ Compared with the OMV-negative *P. gingivalis* mutant, the wild type strengthens antigenicity through OMVs.^38^ First, *P. gingivalis* OMVs increase the surface area to increase antigenicity.^38^ In addition, they retain the immunodominant determinants through LPS and A-LPS modifications, which are more centralized than those in bacteria are, making OMVs more suitable for vaccine development.^38^, ^241^ The intranasal administration of *P. gingivalis* OMVs with the immune adjuvant poly (I:C) induces the secretion of *P. gingivalis*-specific antibodies in the blood and saliva in a dose-dependent manner. In addition to IgG and IgA in serum, secretory IgA (s-IgA) is also induced in nasal rinses and saliva in this way and thus stimulates both haematal and mucosal immune responses (**Figure 6B**).^82^ Furthermore, since s-IgA is usually more cross-reactive than other classes of immunoglobulins are, intranasal immunization with *P. gingivalis* OMVs + poly (I:C) enhances resistance to bacterial variants, which has important implications for vaccine strategies for periodontitis.^242^ In addition to inducing antibodies, intranasal immunization with *P. gingivalis* OMVs + poly (I:C) also inhibits the activity of *P. gingivalis.*^82^ In a mouse model of oral infection, *P. gingivalis* OMVs + poly (I:C) immune serum apparently reduced* P. gingivalis* in the oral cavity compared with that in poly(I:C) mock-immunized mice.^82^ Moreover, the application of *P. gingivalis* OMVs as vaccines can be stable and safe. Owing to their resistance to proteinase K, OMVs are able to withstand long-term storage and stably reach the site of action.^82^ The administered* P. gingivalis* OMVs barely accumulated in proximal organ samples, suggesting that a low dose of* P. gingivalis* OMVs can be safe.^82^ In addition to* P. gingivalis* OMVs,* T. denticola* OMVs can also be used in periodontitis vaccines. Analysis of the protein components and localization of *T. denticola* OMVs can provide new targets for periodontitis vaccine development.^243^ Research on OMVs rich in virulence factors rather than complex bacteria has facilitated vaccine development as well.

Periodontitis arises from the interplay of multiple periodontal pathogens, and different periodontal pathogens produce distinctive OMVs.^182^ It is speculated that a composite multivalent OMV could improve immune efficacy. This conjecture has been preliminarily validated. Intranasal immunization with *P. gingivalis* OMVs alone has relatively low mucosal immunogenicity, whereas the addition of *A. actinomycetemcomitans* OMVs as an immune adjuvant markedly enhances the *P. gingivalis*-specific response, resulting in the production of more serum IgG and salivary IgA and the aggregation of* P. gingivalis* and *A. actinomycetemcomitans* after infection.^145^ In an *in vivo* experiment, mice were intranasally immunized with *P. gingivalis* OMVs + *A. actinomycetemcomitans* OMVs or *P. gingivalis* OMVs + poly (I:C). Then, they were orally administered *P. gingivalis* and *A. actinomycetemcomitans*. Both groups presented a decrease in the number of these bacteria, but the group treated with *A. actinomycetemcomitans* OMVs as an adjuvant presented superior results to the group treated with poly (I:C), demonstrating that this bivalent OMV vaccine can be more effective in preventing periodontitis (**Figure 6B**).^145^ In addition, it is safe, with no serious side effects detected in an intracerebrally injected mouse model.^145^

OMVs have great potential in treating and preventing periodontitis. They can function as drug targets or be employed as antigens and immune adjuvants in periodontitis vaccines. In the field of immunoprophylaxis, *P. gingivalis* OMVs, as vaccine antigens, induce serum antibody production to resist bacterial invasion. However, in previous studies, they were reported to inhibit serum antimicrobial activity against* C. ochracea* and instead protect bacteria. This discrepancy might result from the administration mode and action period. Intranasal immunization with *P. gingivalis* OMVs induces s-IgA production, which elicits immunization of the local nasal cavity and remote mucosal sites to increase the degree of the immune response. However, infection with *P. gingivalis* OMVs does not have this effect. In addition, the persistent period of periodontitis after administration may be another factor. Immunoprophylaxis in healthy periodontal tissue occurs in a different environment than that after periodontitis develops. Different amounts of pathogens and proinflammatory factors may interfere with the specific effects of OMVs. Therefore, the action environment needs to be evaluated first when OMV-related vaccines are used. For example, when bacteria have already destroyed the periodontium, OMVs should be used with caution to prevent further immune escape.

Moreover, considering the virulence factors of OMVs, they should be artificially modified in vaccine development to reduce their virulence and enhance their antigenicity. The systemic effects of these periodontal vaccines should also be taken into account before their clinical use. In addition, OMV vaccines with OMV immune adjuvants have promising therapeutic effects, as the use of *A. actinomycetemcomitans* OMVs increases the degree of the mucosal immune response induced by *P. gingivalis* OMVs. The development of multivalent periodontitis vaccines with multiple OMVs can increase immunoprophylactic ability and effectively eliminate pathogens in periodontitis.

### 5.2 Therapeutic potential of OMVs in OSCC

As OMVs are biocompatible and target specific, they can act as good drug carriers to decrease the degree of lymphatic metastasis in OSCC (**Table 2**).^244^ 5-Fluorouracil (5-FU) is a commonly used antimetabolite and antitumour drug that can inhibit thymidylate synthase activity.^245^, ^246^ It has been widely applied in the treatment of many types of cancers, including OSCC.^247^ However, since OSCC cells acquire 5-FU resistance, more effective therapeutic strategies are needed.^248^ Researchers have modified* E. coli* OMVs to establish a biofilm drug delivery system (OMV-MSN-5-FU) and added it to artificial gastric and artificial intestinal fluids. OMVs have been found to prolong the duration of drug treatment and enhance therapeutic efficacy compared with MSNs-5-FU alone.^244^ Coculturing OSCC cells with OMV-MSN-5-FU demonstrated that OMV-MSN-5-FU can significantly inhibit the proliferative activity of OSCC cells and prevent cancer progression.^244^ In a tumour-loaded mouse model prepared from the human tongue squamous carcinoma cell line Tca8113, OMV-MSN-5-FU injection decreased the level of N-α-acetyltransferase (Naa10), which promotes the proliferation of tumour cells as a progression biomarker.^244^, ^249^, ^250^ OMVs-MSN-5-FU can also regulate the ratio of effector T cells and helper T cells to assist in maintaining stability in the immune environment (**Figure 6C**).^244^ Titanium dioxide (TiO_2_) can produce reactive oxygen species under light or radiation, causing damage to cell membrane, mitochondrion and DNA of cancer cells, which has broad prospects in enhancing sensitivity in photodynamic therapy and radiotherapy of OSCC.^251^, ^252^ However, aqueous-phase TiO_2_ nanoparticles tend to aggregate and that limits their efficacy.^253^ Encapsulating TiO_2_ with *E. coli* OMVs can transform the aqueous-phase TiO_2_ into the oil-phase, which not only enhances the efficacy of TiO_2_ but also enables it to carry tumour antigens and improve the targeting ability through bioengineering, stimulating antitumour immune responses.^254^ The combination of TiO_2_ and *E. coli* OMVs serves as a novel low-dose radiotherapy delivery system TiO_2_@OMV, exerting dual effects of radiosensitization and immune activation (**Figure 6C**). 254 Moreover, virulence factor sRNA23392 in* P*.* gingivalis* OMVs can promote the migration and invasion of OSCC, and its inhibitors can attenuate this process.^217^ Therefore, in *P*.* gingivalis* related OSCC, the sRNA23392 inhibitors can be applied to inhibit the metastasis of OSCC by targeting *P*.* gingivalis* OMVs (**Figure 6C**). Overall, OMVs are promising drug delivery agents and drug targets for OSCC therapy.

Recently, the application of OMVs as immune stimulators in cancer immunotherapy has received much attention.^83^, ^89^, ^90^ Considering that bioengineered OMVs can be loaded with tumour antigens in cancer immunotherapy, they have the potential to be modified to express multiple OSCC-associated antigens on the surface and strongly activate antitumour immunity, thereby reversing OSCC progression. Some OSCC-associated antigens, such as melanoma-associated antigens and MUC1, are attractive targets for OSCC immunotherapy, but whether these antigens are suitable for expression on OMVs and how to display them on the surface of OMVs by bioengineering remain to be explored.^255^, ^256^ In addition, identifying additional OSCC-associated antigens to increase the immunostimulatory effects of bioengineered OMVs is equally important.

Moreover, as the occurrence and progression of OSCC are closely related to OMVs from oral pathogenic bacteria, the removal of OMVs in bacteria-related OSCC treatment is also particularly important. Similar to therapies for periodontitis, drugs that target OMVs can be developed to eliminate them. For example, since HEF and curcumin target *P. gingivalis* OMVs to inhibit periodontitis progression, experiments can use these drugs in OMV-related OSCC to explore whether they can also target *P. gingivalis* OMVs under these conditions.

## 6. Conclusion and perspectives

OMVs are vesicles derived from the OM of gram-negative bacteria through OM blebbing and explosive cell lysis. The contents encompass signalling molecules and virulence factors, including various proteins, lipids, nucleic acids, and peptidoglycans, through which OMVs interact with bacteria and host cells. OMVs of probiotics can benefit host health, while OMVs of oral pathogens play a pathogenic role in periodontitis and OSCC, accelerating the development of oral diseases.

OMVs from the periodontitis "red complex" consortium (*P. gingivalis*, *T. forsythia*, and *T. denticola*) and *A. actinomycetemcomitans* have drawn considerable attention. The virulence factors and functions of these bacteria under different conditions have been widely studied.^257^, ^258^ However, OMVs from other oral gram-negative pathogens, such as *C. ochracea* and *F. nucleatum*, have not been extensively studied. Although these bacteria seem subordinate in the pathogenesis of periodontitis, their OMVs may also cause severe damage to the periodontium by promoting biofilm formation and supporting other important pathogens. For example, *C. ochracea* participates in early plaque formation, and its OMVs are likely involved in the early colonization of *C. ochracea* and biofilm formation in cooperation with other bacteria. Controlling the production of *C. ochracea* OMVs may help to inhibit the formation of pathogenic biofilms. Therefore, it is necessary to explore the contents and mechanisms of OMVs from these “unpopular” pathogenic bacteria. *F. nucleatum* OMVs can independently exacerbate periodontitis by enhancing inflammation of the periodontium and the absorption of alveolar bone.^13^ The interaction between *F. nucleatum* OMVs and HPDLSCs triggers the activation of the NLRP3 inflammasome and a decrease in the accumulation of mineralization in HPDLSCs, which ultimately leads to damage to the periodontium and resorption of alveolar bone.

Notably, oral bacteria play an important role in systemic diseases, and OMVs represent their main virulence and increase their ability to diffuse, which is an important way for oral bacteria to spread throughout the whole body. The systemic effects of *P. gingivalis* OMVs have been extensively studied. They can indirectly affect the body by promoting the pathogenicity of other pathogens, such as *S. aureus* and HIV-1. *P. gingivalis* OMVs themselves also directly influence the CNS, circulatory system, endocrine system and immune system, such as by inducing neuroinflammation, increasing vascular leakage, reducing insulin sensitivity and promoting the RA process. The potential damaging effects of *T. denticola* OMVs and *A. actinomycetemcomitans* OMVs in the CNS have also been suggested. These results suggest that the OMVs of oral bacteria are a vital source of many systemic diseases. However, current studies on this topic, especially the systemic action of non-*P. gingivalis* OMVs, are insufficient. It is necessary to focus on the systemic actions and mechanisms of oral bacteria and their OMVs to further explore the role of various oral OMVs and to develop biotherapies for related systemic diseases.

In addition to their pathogenic effects, OMVs also have therapeutic importance in periodontitis and OSCC. They can act as drug carriers and immune stimulators, such as vaccines and adjuvants. However, its clinical application is still a long way off. The balance between reducing the toxicity and maintaining the antigenicity of OMVs from pathogenic bacteria and the improvement of their targeting ability are important challenges of the OMV therapy.^259^ Meanwhile, their proinflammatory properties may cause the risk of inflammation throughout the body.^260^ Bioengineering of OMVs is a powerful approach to addressing these issues existing in natural OMVs. However, in the field of stomatology, research on bioengineering of OMVs is still very scarce. More studies are required to overcome the existing limitations of OMVs in order to apply them in clinical treatment. Bioengineering of OMVs involves the modification of bacteria before OMV isolation or direct modification of the OMV properties after isolation.^259^ For the modification before OMV isolation, through deletion or modification of the bacterial genes, the toxins and unwanted antigens in the mutant-derived OMVs can then be removed, and the specific homologous and heterologous antigens are expressed, thereby balancing safety and antigenicity. For example, OMVs are derived from gram-negative bacteria, and the LPS on their surface is a potent TLR4 agonist that enhances the immune response. However, as LPS is also toxic to the body and may lead to excessive inflammatory responses, the expression of LPS needs to be moderately reduced in the OMV vaccine. A study conducted gene editing on *Salmonella Typhimurium* by constructing plasmids, deleting *fljB*, *eptA*, *arnT* and *lpxT*, and introducing lxpE to construct the targeted mutant strain, modifying LPS to monophosphoryl lipid A.^261^ Compared with OMVs from the wild type, those from the mutant significantly reduced the degree of organ damage and decreased the production of proinflammatory factors throughout the body. 261 Bioengineering of OMVs from probiotics can also be promising. Modifying the genes of probiotics to express the heterologous protein of pathogens on the OMVs may be a strategy to enhance the bioactivity as well as guarantee the safety.^262^ For the modification after OMV isolation, the molecules are directly modified on the surface of the OMVs or inside, which is more flexible for it can modify more kinds of molecules in various ways like chemical coupling and membrane fusion.^90^, ^263^-^265^ While the bioengineered OMVs still have application challenges. Notably, the quantity of OMVs produced varies under different conditions. Whether modification of the antigens will influence the quantity of OMVs and how bioengineering can help to improve the quantity as well as ensure the safety and antigenicity needs further research.^266^

At present, there is still a lack of direct evidence to prove that oral OMVs play a role in the prevention and treatment of systemic diseases. But existing studies have shown that they can spread oral diseases such as periodontitis and OSCC throughout the body and take part in treating these diseases. This suggests that oral OMVs have a potential indirect therapeutic effect on some odontogenic systemic diseases. Experiments can be designed to explore the systemic effects of OMV therapy in oral diseases, in order to further study the application prospects of oral OMVs in the prevention and treatment of relevant systemic diseases.

Most studies on the role of OMVs in oral diseases have focused on pathogenic OMVs, whereas little attention has been given to OMVs from nonpathogenic oral bacteria. Considering that OMVs from probiotics have been found to be beneficial to the body and can be used as safe and efficient drug carriers in intestinal diseases, oral probiotic OMVs are also worthy of investigation. The use of probiotic OMVs as drug and antigen carriers in periodontitis and OSCC is safer and more beneficial to oral health than the use of pathogenic OMVs. Researchers have employed nonpathogenic *E. coli* Nissle 1917 strain to carry antigens in an oral vaccine and found that it has the potential to induce considerable systemic and mucosal humoral reactions. However, the effects are not stable enough and the colonization ability of bacteria is weak.^267^ In contrast, with strong adhesion and strong involvement in biofilm formation, probiotic OMVs may improve colonization ability and represent a better choice for antigen carriers. OMVs from other nonpathogenic gram-negative bacteria in the oral cavity may also carry some characteristics of the parent bacteria and exert beneficial effects on oral health beyond the role of vaccine carriers.

In conclusion, oral OMVs play critical pathogenic roles in periodontitis and OSCC, with strong effects on the spread of systemic diseases. They are also promising candidates for disease prevention and treatment as therapeutic drug targets, vaccines, and drug carriers. Oral OMVs, as a broad group, have gained increasing attention in recent years, and more in-depth research is needed to elucidate their roles in pathogenesis and treatment. The application of oral OMVs in therapies for oral diseases and related systemic diseases will be a major direction for future experiments.

## Figures and Tables

**Table 1:** OMVs involved in oral diseases

No.	Diseases	OMVs	Key factors	Functions	Ref.
1.	Periodontitis	*P. gingivalis* OMVs	Gingipains (active)	Degrading bactericidal granule-derived proteins and peptides like MPO and LL-37 and preventing the bacteria to be killed by neutrophils in the gingival crevice	102
			Gingipains (inactive)	Activating the pro-inflammatory immune response in human telomerase-immortalized gingival keratinocytes	189
				Stimulating the AKT pathway in dendritic cells in periodontal tissues and leading to alveolar bone resorption	189
			PPAD	Promoting the OMV biogenesis and the systemic effects	41
			msRNA	Targeting the apoptosis-related gene Chromobox 5 to promote the apoptosis of HPDLCs	108
			LPS	Interacting with PRRs and causing chronic inflammatory dysregulation	111
			SL	Inhibiting the immune response of the THP-1 cells	114
		*T. forsythia* OMVs	Miropin	Causing damage to periodontal support structures by inhibiting degrading fibrin deposits	200
		*T. denticola* OMVs	Msp	Promoting the OMV biogenesis	137
				Restricting PIP signaling, disrupting the phagocytose function of neutrophils, and helping the bacteria to evade host immune responses	139
			LOS	Increasing OMVs' adhesion to periodontal tissues	201
				Promoting *P. micros* to secret pro-inflammatory cytokines	201
			Dentilisin	Degrading the tight junctional proteins to disrupt the cellular tight junctions in periodontal epitheliums	68
		*A. actinomycetemcomitans* OMVs	LtxA	Killing defense cells to protect the bacteria from phagocytosis	146-148, 152
			CDT	Cleaving the DNA and leading to a rapid growth arrest in susceptible cells of the periodontium	26
		*F. nucleatum* OMVs	Major OM protein	Taking part in immunomodulation and adhesion by making M0-like macrophages tend to be differentiated into the M1 phenotype	165
		C. ochracea OMVs	Unsaturated fatty acid of phosphatidylinositol	Promoting the OMV biogenesis	168
			Microbial EET	Influencing the metabolism of other oral bacteria through EET	169
2.	OSCC	*P. gingivalis* OMVs	sRNA23392	Targeting DSC2 to accelerate the invasion and migration of the cancer	217
		*F. nucleatum* OMVs	EMT	Promoting lung metastasis of OSCC through EMT	12

**Table 2:** Therapeutic effects of OMVs in oral diseases

No.	Diseases	Roles	OMVs	Drugs/immune adjuvants	Effects of the drugs/immune adjuvants	Ref.
1.	Periodontitis	Drug target	*P. gingivalis* OMVs	HEF	Inducing bacterial disintegration, overproducing OMVs with aberrant surface properties and leading to the rapid death of P.* gingivalis*	78
				Curcumin	Suppressing the OMV-mediated expression of IL-6, IL-1β, and TNF-α in HGECs	79
					Inhibiting OMVs' adhesion to gingival epithelial cells and restraining their toxic effects on cell migration	79
			*T. forsythia* OMVs	QSI	Decreasing the OMV-induced production of TNF-α, IL- 1β, IL-6 and IL-8 in THP-1 monocytes	235
		Antigen and immune adjuvant in vaccine	*P. gingivalis* OMVs	Poly(I:C)	Effectively inducing serum IgG and IgA, and mucosal secretory IgA	82
				*A. actinomycetemcomitans* OMVs	More potent than Poly(I:C) as the immune adjuvant	145
2.	OSCC	Drug carrier	*E. coli* OMVs	OMV-MSN-5-FU delivery system	Prolonging the duration of action and enhancing the therapeutic efficacy	244
				TiO_2_@OMV delivery system	Exerting dual effects of radiosensitization and immune activation	254
		Drug target	*P. gingivalis* OMVs	sRNA23392 inhibitors	Inhibiting the *P. gingivalis* OMV-related metastasis of OSCC	217

## Abbreviations

*E. coli*: *Escherichia coli*
*P. gingivalis*: *Porphyromonas gingivalis*
*C. ochracea*: *Capnocytophaga ochracea*
*T. denticola*: *Treponema denticola*
*F. nucleatum*: *Fusobacterium nucleatum*
*T. forsythia*: *Tannerella forsythia*
*A. actinomycetemcomitans*: *Aggregatibacter actinomycetemcomitans*
*S. aureus*: *Staphylococcus aureus*
EVs: extracellular vesicles
BEVs: bacterial extracellular vesicles
OMVs: outer membrane vesicles
OIMVs: outer inner membrane vesicles
TSMSs: tube-shaped membranous structures
CMVs: cytoplasmic membrane vesicles
PRRs: pattern recognition receptors
SL: sphingolipid
TLR: Toll-like receptor
Msp: major sheath protein
LOS: lipooligosaccharide
TER: transepithelial resistance
HEp-2: human epithelial type 2
SHIP1: Src homology 2 domain-containing inositol phosphatase 1
PTEN: phosphatase and tensin homologue
PI3K: phosphatidylinositol 3-kinase
PIP: phosphatidylinositol phosphate
NOD: nucleotide-binding oligomerization domain
BMDMs: bone marrow-derived macrophages
OM: outer membrane
OSCC: oral squamous cell carcinoma
LtxA: leukotoxin A
CDT: cytolethal distending toxin
LPS: lipopolysaccharide
PAMPs: pathogen-associated molecular patterns
RA: rheumatoid arthritis
CNS: central nervous system
AD: Alzheimer's disease
HEF: N-hexane-extracted fennel
siRNAs: small interfering RNAs
TNF: tumour necrosis factor
BMDCs: bone marrow-derived dendritic cells
Rgp: Arg-gingipain
Kgp: Lys-gingipain
T9SS: the type IX secretion system
A-LPS: anionic lipopolysaccharide
CTD: C-terminal structural domain
MPO: myeloperoxidase
PPAD: Peptidylarginine deiminase
msRNAs: microRNA-sized small RNAs
HPDLCs: human periodontal ligament cells
CBX5: Chromobox 5
Bcl-2: B-cell lymphoma-2
NLRP3: pyrin domain containing 3
MGFs: mouse gingival fibroblasts
HPDLSCs: human periodontal ligament stem cells
PI: phosphatidylinositol
EET: extracellular electron transfer
HGECs: human gingival epithelial cells
EGFR: epidermal growth factor receptor
TIGK: telomerase-immortalized gingival keratinocyte
DSC2: Desmocollin 2
EMT: epithelial-mesenchymal transition
CHQ: chloroquine
PECAM-1: platelet endothelial cell adhesion molecule 1
HMEC-1: human microvascular endothelial cells
VSMCs: vascular smooth muscle cells
GSK-3β: glycogen synthase kinase-3 β
HMC3: human microglial clone 3
SAB: *Staphylococcus aureus* bacteraemia
BBB: blood‒brain barrier
QS: Quorum sensing
QSIs: Quorum-sensing inhibitors
5-FU: 5-Fluorouracil
Naa10: N-α-acetyltransferase
TiO_2_: Titanium dioxide

## References

[1] van Niel G, D'Angelo G, Raposo G (2018). Shedding light on the cell biology of extracellular vesicles. Nat Rev Mol Cell Biol.

[2] Kalluri R, LeBleu VS (2020). The biology, function, and biomedical applications of exosomes. Science.

[3] Juodeikis R, Carding SR (2022). Outer Membrane Vesicles: Biogenesis, Functions, and Issues. Microbiol Mol Biol Rev.

[4] Toyofuku M, Nomura N, Eberl L (2019). Types and origins of bacterial membrane vesicles. Nat Rev Microbiol.

[5] Bishop DG, Work E (1965). An extracellular glycolipid produced by Escherichia coli grown under lysine-limiting conditions. Biochem J.

[6] Taylor A, Knox KW, Work E (1966). Chemical and biological properties of an extracellular lipopolysaccharide from Escherichia coli grown under lysine-limiting conditions. Biochem J.

[7] Roier S, Zingl FG, Cakar F, Schild S (2016). Bacterial outer membrane vesicle biogenesis: a new mechanism and its implications. Microb Cell.

[8] Higham SL, Baker S, Flight KE, Krishna A, Kellam P, Reece ST (2023). Intranasal immunization with outer membrane vesicles (OMV) protects against airway colonization and systemic infection with Acinetobacter baumannii. J Infect.

[9] Zingl FG, Kohl P, Cakar F, Leitner DR, Mitterer F, Bonnington KE (2020). Outer Membrane Vesiculation Facilitates Surface Exchange and In Vivo Adaptation of Vibrio cholerae. Cell Host Microbe.

[10] Li J, Sun M, Liu L, Yang W, Sun A, Yu J (2023). Nanoprobiotics for Remolding the Pro-inflammatory Microenvironment and Microbiome in the Treatment of Colitis. Nano Lett.

[11] Gabarrini G, Grasso S, van Winkelhoff AJ, van Dijl JM (2020). Gingimaps: Protein Localization in the Oral Pathogen Porphyromonas gingivalis. Microbiol Mol Biol Rev.

[12] Chen G, Gao C, Jiang S, Cai Q, Li R, Sun Q (2023). Fusobacterium nucleatum outer membrane vesicles activate autophagy to promote oral cancer metastasis. J Adv Res.

[13] Zhang L, Zhang D, Liu C, Tang B, Cui Y, Guo D (2024). Outer Membrane Vesicles Derived From Fusobacterium nucleatum Trigger Periodontitis Through Host Overimmunity. Adv Sci (Weinh).

[14] Deatherage BL, Lara JC, Bergsbaken T, Rassoulian Barrett SL, Lara S, Cookson BT (2009). Biogenesis of bacterial membrane vesicles. Mol Microbiol.

[15] Turnbull L, Toyofuku M, Hynen AL, Kurosawa M, Pessi G, Petty NK (2016). Explosive cell lysis as a mechanism for the biogenesis of bacterial membrane vesicles and biofilms. Nat Commun.

[16] Orench-Rivera N, Kuehn MJ (2016). Environmentally controlled bacterial vesicle-mediated export. Cell Microbiol.

[17] Mashburn-Warren LM, Whiteley M (2006). Special delivery: vesicle trafficking in prokaryotes. Mol Microbiol.

[18] Roier S, Zingl FG, Cakar F, Durakovic S, Kohl P, Eichmann TO (2016). A novel mechanism for the biogenesis of outer membrane vesicles in Gram-negative bacteria. Nat Commun.

[19] Kulp A, Kuehn MJ (2010). Biological functions and biogenesis of secreted bacterial outer membrane vesicles. Annu Rev Microbiol.

[20] Cooke AC, Florez C, Dunshee EB, Lieber AD, Terry ML, Light CJ (2020). Pseudomonas Quinolone Signal-Induced Outer Membrane Vesicles Enhance Biofilm Dispersion in Pseudomonas aeruginosa. mSphere.

[21] Aschtgen MS, Lynch JB, Koch E, Schwartzman J, McFall-Ngai M, Ruby E (2016). Rotation of Vibrio fischeri Flagella Produces Outer Membrane Vesicles That Induce Host Development. J Bacteriol.

[22] Catalão MJ, Gil F, Moniz-Pereira J, São-José C, Pimentel M (2013). Diversity in bacterial lysis systems: bacteriophages show the way. FEMS Microbiol Rev.

[23] Manning AJ, Kuehn MJ (2011). Contribution of bacterial outer membrane vesicles to innate bacterial defense. BMC Microbiol.

[24] Nakao R, Takashiba S, Kosono S, Yoshida M, Watanabe H, Ohnishi M (2014). Effect of Porphyromonas gingivalis outer membrane vesicles on gingipain-mediated detachment of cultured oral epithelial cells and immune responses. Microbes Infect.

[25] Nice JB, Balashova NV, Kachlany SC, Koufos E, Krueger E, Lally ET (2018). Aggregatibacter actinomycetemcomitans Leukotoxin Is Delivered to Host Cells in an LFA-1-Indepdendent Manner When Associated with Outer Membrane Vesicles. Toxins (Basel).

[26] Rompikuntal PK, Thay B, Khan MK, Alanko J, Penttinen AM, Asikainen S (2012). Perinuclear localization of internalized outer membrane vesicles carrying active cytolethal distending toxin from Aggregatibacter actinomycetemcomitans. Infect Immun.

[27] Eletto D, Mentucci F, Voli A, Petrella A, Porta A, Tosco A (2022). Helicobacter pylori Pathogen-Associated Molecular Patterns: Friends or Foes?. Int J Mol Sci.

[28] Ellis TN, Kuehn MJ (2010). Virulence and immunomodulatory roles of bacterial outer membrane vesicles. Microbiol Mol Biol Rev.

[29] Renelli M, Matias V, Lo RY, Beveridge TJ (2004). DNA-containing membrane vesicles of Pseudomonas aeruginosa PAO1 and their genetic transformation potential. Microbiology (Reading).

[30] Okamura H, Hirota K, Yoshida K, Weng Y, He Y, Shiotsu N (2021). Outer membrane vesicles of Porphyromonas gingivalis: Novel communication tool and strategy. Jpn Dent Sci Rev.

[31] Johnston EL, Zavan L, Bitto NJ, Petrovski S, Hill AF, Kaparakis-Liaskos M (2023). Planktonic and Biofilm-Derived Pseudomonas aeruginosa Outer Membrane Vesicles Facilitate Horizontal Gene Transfer of Plasmid DNA. Microbiol Spectr.

[32] Lieberman LA (2022). Outer membrane vesicles: A bacterial-derived vaccination system. Front Microbiol.

[33] Choi JW, Kim SC, Hong SH, Lee HJ (2017). Secretable Small RNAs via Outer Membrane Vesicles in Periodontal Pathogens. J Dent Res.

[34] Jha C, Ghosh S, Gautam V, Malhotra P, Ray P (2017). In vitro study of virulence potential of Acinetobacter baumannii outer membrane vesicles. Microb Pathog.

[35] Jang SC, Kim SR, Yoon YJ, Park KS, Kim JH, Lee J (2015). In vivo kinetic biodistribution of nano-sized outer membrane vesicles derived from bacteria. Small.

[36] Seyama M, Yoshida K, Yoshida K, Fujiwara N, Ono K, Eguchi T (2020). Outer membrane vesicles of Porphyromonas gingivalis attenuate insulin sensitivity by delivering gingipains to the liver. Biochim Biophys Acta Mol Basis Dis.

[37] Martinez-Martinez RE, Abud-Mendoza C, Patiño-Marin N, Rizo-Rodríguez JC, Little JW, Loyola-Rodríguez JP (2009). Detection of periodontal bacterial DNA in serum and synovial fluid in refractory rheumatoid arthritis patients. J Clin Periodontol.

[38] Nakao R, Hasegawa H, Ochiai K, Takashiba S, Ainai A, Ohnishi M (2011). Outer membrane vesicles of Porphyromonas gingivalis elicit a mucosal immune response. PLoS One.

[39] Mantri CK, Chen CH, Dong X, Goodwin JS, Pratap S, Paromov V (2015). Fimbriae-mediated outer membrane vesicle production and invasion of Porphyromonas gingivalis. Microbiologyopen.

[40] Ho MH, Chen CH, Goodwin JS, Wang BY, Xie H (2015). Functional Advantages of Porphyromonas gingivalis Vesicles. PLoS One.

[41] du Teil Espina M, Haider Rubio A, Fu Y, López-Álvarez M, Gabarrini G, van Dijl JM (2022). Outer membrane vesicles of the oral pathogen Porphyromonas gingivalis promote aggregation and phagocytosis of Staphylococcus aureus. Front Oral Health.

[42] Dong XH, Ho MH, Liu B, Hildreth J, Dash C, Goodwin JS (2018). Role of Porphyromonas gingivalis outer membrane vesicles in oral mucosal transmission of HIV. Sci Rep.

[43] Kadurugamuwa JL, Beveridge TJ (1996). Bacteriolytic effect of membrane vesicles from Pseudomonas aeruginosa on other bacteria including pathogens: conceptually new antibiotics. J Bacteriol.

[44] Williams D, Vicôgne J, Zaitseva I, McLaughlin S, Pessin JE (2009). Evidence that electrostatic interactions between vesicle-associated membrane protein 2 and acidic phospholipids may modulate the fusion of transport vesicles with the plasma membrane. Mol Biol Cell.

[45] Furuta N, Tsuda K, Omori H, Yoshimori T, Yoshimura F, Amano A (2009). Porphyromonas gingivalis outer membrane vesicles enter human epithelial cells via an endocytic pathway and are sorted to lysosomal compartments. Infect Immun.

[46] Bomberger JM, Maceachran DP, Coutermarsh BA, Ye S, O'Toole GA, Stanton BA (2009). Long-distance delivery of bacterial virulence factors by Pseudomonas aeruginosa outer membrane vesicles. PLoS Pathog.

[47] Fulsundar S, Harms K, Flaten GE, Johnsen PJ, Chopade BA, Nielsen KM (2014). Gene transfer potential of outer membrane vesicles of Acinetobacter baylyi and effects of stress on vesiculation. Appl Environ Microbiol.

[48] Sartorio MG, Pardue EJ, Feldman MF, Haurat MF (2021). Bacterial Outer Membrane Vesicles: From Discovery to Applications. Annu Rev Microbiol.

[49] Lee EY, Bang JY, Park GW, Choi DS, Kang JS, Kim HJ (2007). Global proteomic profiling of native outer membrane vesicles derived from Escherichia coli. Proteomics.

[50] Biller SJ, Schubotz F, Roggensack SE, Thompson AW, Summons RE, Chisholm SW (2014). Bacterial vesicles in marine ecosystems. Science.

[51] Li Z, Clarke AJ, Beveridge TJ (1998). Gram-negative bacteria produce membrane vesicles which are capable of killing other bacteria. J Bacteriol.

[52] Beveridge TJ (1999). Structures of gram-negative cell walls and their derived membrane vesicles. J Bacteriol.

[53] Kadurugamuwa JL, Mayer A, Messner P, Sára M, Sleytr UB, Beveridge TJ (1998). S-layered Aneurinibacillus and Bacillus spp. are susceptible to the lytic action of Pseudomonas aeruginosa membrane vesicles. J Bacteriol.

[54] Kadurugamuwa JL, Beveridge TJ (1995). Virulence factors are released from Pseudomonas aeruginosa in association with membrane vesicles during normal growth and exposure to gentamicin: a novel mechanism of enzyme secretion. J Bacteriol.

[55] Mashburn LM, Whiteley M (2005). Membrane vesicles traffic signals and facilitate group activities in a prokaryote. Nature.

[56] Inagaki S, Onishi S, Kuramitsu HK, Sharma A (2006). Porphyromonas gingivalis vesicles enhance attachment, and the leucine-rich repeat BspA protein is required for invasion of epithelial cells by "Tannerella forsythia". Infect Immun.

[57] Schooling SR, Beveridge TJ (2006). Membrane vesicles: an overlooked component of the matrices of biofilms. J Bacteriol.

[58] Güler G, Vorob'ev MM, Vogel V, Mäntele W (2016). Proteolytically-induced changes of secondary structural protein conformation of bovine serum albumin monitored by Fourier transform infrared (FT-IR) and UV-circular dichroism spectroscopy. Spectrochim Acta A Mol Biomol Spectrosc.

[59] Schwechheimer C, Kuehn MJ (2015). Outer-membrane vesicles from Gram-negative bacteria: biogenesis and functions. Nat Rev Microbiol.

[60] Kinder SA, Holt SC (1993). Localization of the Fusobacterium nucleatum T18 adhesin activity mediating coaggregation with Porphyromonas gingivalis T22. J Bacteriol.

[61] Kamble NS, Thomas S, Madaan T, Ehsani N, Sange S, Tucker K (2025). Engineered bacteria as an orally administered anti-viral treatment and immunization system. Gut Microbes.

[62] Chai QQ, Li D, Zhang M, Gu YW, Li AX, Wu X (2025). Engineering nanoplatforms of bacterial outer membrane vesicles to overcome cancer therapy resistance. Drug Resist Updat.

[63] Li N, Wang M, Liu F, Wu P, Wu F, Xiao H (2024). Bioorthogonal Engineering of Bacterial Outer Membrane Vesicles for NIR-II Fluorescence Imaging-Guided Synergistic Enhanced Immunotherapy. Anal Chem.

[64] Zhu X, Zou A, Zhao M, Hou J, Xianyu Y (2025). Probiotic Vesicles-Implemented Multifunctional Nanotherapeutic Approach for Antibacterial, Anti-inflammatory, and Tissue Regeneration in Bacterial-Infected Oral Ulcer Healing. Adv Healthc Mater.

[65] Yao Q, Liu T, Wen J, Yang Q, Li Y, Yan H (2025). SpyTag-PEGylated probiotics delivering IL-1Ra modulate gut-lung crosstalk to mitigate septic lung injury. J Control Release.

[66] Ma D, Zhang Y, Zhang J, Shi J, Gao S, Long F (2025). Outer membrane vesicles derived from probiotic Escherichia coli Nissle 1917 promote metabolic remodeling and M1 polarization of RAW264.7 macrophages. Front Immunol.

[67] Hong J, Dauros-Singorenko P, Whitcombe A, Payne L, Blenkiron C, Phillips A (2019). Analysis of the Escherichia coli extracellular vesicle proteome identifies markers of purity and culture conditions. J Extracell Vesicles.

[68] Chi B, Qi M, Kuramitsu HK (2003). Role of dentilisin in Treponema denticola epithelial cell layer penetration. Res Microbiol.

[69] Chen S, Lei Q, Zou X, Ma D (2023). The role and mechanisms of gram-negative bacterial outer membrane vesicles in inflammatory diseases. Front Immunol.

[70] Tiku V, Kofoed EM, Yan D, Kang J, Xu M, Reichelt M (2021). Outer membrane vesicles containing OmpA induce mitochondrial fragmentation to promote pathogenesis of Acinetobacter baumannii. Sci Rep.

[71] Koeppen K, Hampton TH, Jarek M, Scharfe M, Gerber SA, Mielcarz DW (2016). A Novel Mechanism of Host-Pathogen Interaction through sRNA in Bacterial Outer Membrane Vesicles. PLoS Pathog.

[72] Shah B, Sullivan CJ, Lonergan NE, Stanley S, Soult MC, Britt LD (2012). Circulating bacterial membrane vesicles cause sepsis in rats. Shock.

[73] Uemura Y, Hiroshima Y, Tada A, Murakami K, Yoshida K, Inagaki Y (2022). Porphyromonas gingivalis Outer Membrane Vesicles Stimulate Gingival Epithelial Cells to Induce Pro-Inflammatory Cytokines via the MAPK and STING Pathways. Biomedicines.

[74] Corrado C, Raimondo S, Chiesi A, Ciccia F, De Leo G, Alessandro R (2013). Exosomes as intercellular signaling organelles involved in health and disease: basic science and clinical applications. Int J Mol Sci.

[75] Yang WW, Guo B, Jia WY, Jia Y (2016). Porphyromonas gingivalis-derived outer membrane vesicles promote calcification of vascular smooth muscle cells through ERK1/2-RUNX2. FEBS Open Bio.

[76] Yoshida K, Yoshida K, Seyama M, Hiroshima Y, Mekata M, Fujiwara N (2022). Porphyromonas gingivalis outer membrane vesicles in cerebral ventricles activate microglia in mice. Oral Dis.

[77] Gabarrini G, Heida R, van Ieperen N, Curtis MA, van Winkelhoff AJ, van Dijl JM (2018). Dropping anchor: attachment of peptidylarginine deiminase via A-LPS to secreted outer membrane vesicles of Porphyromonas gingivalis. Sci Rep.

[78] Yoshino N, Ikeda T, Nakao R (2022). Dual Inhibitory Activity of Petroselinic Acid Enriched in Fennel Against Porphyromonas gingivalis. Front Microbiol.

[79] Izui S, Sekine S, Murai H, Takeuchi H, Amano A (2021). Inhibitory effects of curcumin against cytotoxicity of Porphyromonas gingivalis outer membrane vesicles. Arch Oral Biol.

[80] Gnopo YMD, Watkins HC, Stevenson TC, DeLisa MP, Putnam D (2017). Designer outer membrane vesicles as immunomodulatory systems - Reprogramming bacteria for vaccine delivery. Adv Drug Deliv Rev.

[81] Bachmann MF, Jennings GT (2010). Vaccine delivery: a matter of size, geometry, kinetics and molecular patterns. Nat Rev Immunol.

[82] Nakao R, Hasegawa H, Dongying B, Ohnishi M, Senpuku H (2016). Assessment of outer membrane vesicles of periodontopathic bacterium Porphyromonas gingivalis as possible mucosal immunogen. Vaccine.

[83] Cheng K, Zhao R, Li Y, Qi Y, Wang Y, Zhang Y (2021). Bioengineered bacteria-derived outer membrane vesicles as a versatile antigen display platform for tumor vaccination via Plug-and-Display technology. Nat Commun.

[84] Gujrati V, Kim S, Kim SH, Min JJ, Choy HE, Kim SC (2014). Bioengineered bacterial outer membrane vesicles as cell-specific drug-delivery vehicles for cancer therapy. ACS Nano.

[85] Chen Y, Gao Y, Chen Y, Liu L, Mo A, Peng Q (2020). Nanomaterials-based photothermal therapy and its potentials in antibacterial treatment. J Control Release.

[86] de Nies L, Kobras CM, Stracy M (2023). Antibiotic-induced collateral damage to the microbiota and associated infections. Nat Rev Microbiol.

[87] Huang W, Meng L, Chen Y, Dong Z, Peng Q (2022). Bacterial outer membrane vesicles as potential biological nanomaterials for antibacterial therapy. Acta Biomater.

[88] Wang S, Huang W, Li K, Yao Y, Yang X, Bai H (2017). Engineered outer membrane vesicle is potent to elicit HPV16E7-specific cellular immunity in a mouse model of TC-1 graft tumor. Int J Nanomedicine.

[89] Qing S, Lyu C, Zhu L, Pan C, Wang S, Li F (2020). Biomineralized Bacterial Outer Membrane Vesicles Potentiate Safe and Efficient Tumor Microenvironment Reprogramming for Anticancer Therapy. Adv Mater.

[90] Zhao X, Zhao R, Nie G (2022). Nanocarriers based on bacterial membrane materials for cancer vaccine delivery. Nat Protoc.

[91] Bae EH, Seo SH, Kim CU, Jang MS, Song MS, Lee TY (2019). Bacterial Outer Membrane Vesicles Provide Broad-Spectrum Protection against Influenza Virus Infection via Recruitment and Activation of Macrophages. J Innate Immun.

[92] Waller T, Kesper L, Hirschfeld J, Dommisch H, Kölpin J, Oldenburg J (2016). Porphyromonas gingivalis Outer Membrane Vesicles Induce Selective Tumor Necrosis Factor Tolerance in a Toll-Like Receptor 4- and mTOR-Dependent Manner. Infect Immun.

[93] Lim Y, Kim HY, An SJ, Choi BK (2022). Activation of bone marrow-derived dendritic cells and CD4(+) T cell differentiation by outer membrane vesicles of periodontal pathogens. J Oral Microbiol.

[94] Perricone C, Ceccarelli F, Saccucci M, Di Carlo G, Bogdanos DP, Lucchetti R (2019). Porphyromonas gingivalis and rheumatoid arthritis. Curr Opin Rheumatol.

[95] Reyes L (2021). Porphyromonas gingivalis. Trends Microbiol.

[96] Veith PD, Glew MD, Gorasia DG, Cascales E, Reynolds EC (2022). The Type IX Secretion System and Its Role in Bacterial Function and Pathogenesis. J Dent Res.

[97] Gui MJ, Dashper SG, Slakeski N, Chen YY, Reynolds EC (2016). Spheres of influence: Porphyromonas gingivalis outer membrane vesicles. Mol Oral Microbiol.

[98] Veith PD, Chen YY, Chen D, O'Brien-Simpson NM, Cecil JD, Holden JA (2015). Tannerella forsythia Outer Membrane Vesicles Are Enriched with Substrates of the Type IX Secretion System and TonB-Dependent Receptors. J Proteome Res.

[99] Haurat MF, Aduse-Opoku J, Rangarajan M, Dorobantu L, Gray MR, Curtis MA (2011). Selective sorting of cargo proteins into bacterial membrane vesicles. J Biol Chem.

[100] Veith PD, Chen YY, Gorasia DG, Chen D, Glew MD, O'Brien-Simpson NM (2014). Porphyromonas gingivalis outer membrane vesicles exclusively contain outer membrane and periplasmic proteins and carry a cargo enriched with virulence factors. J Proteome Res.

[101] Lasica AM, Ksiazek M, Madej M, Potempa J (2017). The Type IX Secretion System (T9SS): Highlights and Recent Insights into Its Structure and Function. Front Cell Infect Microbiol.

[102] du Teil Espina M, Fu Y, van der Horst D, Hirschfeld C, López-Álvarez M, Mulder LM (2022). Coating and Corruption of Human Neutrophils by Bacterial Outer Membrane Vesicles. Microbiol Spectr.

[103] Vitkov L, Muñoz LE, Schoen J, Knopf J, Schauer C, Minnich B (2021). Neutrophils Orchestrate the Periodontal Pocket. Front Immunol.

[104] Xia X (2023). Fabrication of CdS quantum dots with egg white and application in the assay of hypochlorous acid and myeloperoxidase activity and inhibition. Anal Methods.

[105] McCrudden MT, Orr DF, Yu Y, Coulter WA, Manning G, Irwin CR (2013). LL-37 in periodontal health and disease and its susceptibility to degradation by proteinases present in gingival crevicular fluid. J Clin Periodontol.

[106] Pastuszak K, Kowalczyk B, Tarasiuk J, Luchowski R, Gruszecki WI, Jurak M (2023). Insight into the Mechanism of Interactions between the LL-37 Peptide and Model Membranes of Legionella gormanii Bacteria. Int J Mol Sci.

[107] Vermilyea DM, Moradali MF, Kim HM, Davey ME (2021). PPAD Activity Promotes Outer Membrane Vesicle Biogenesis and Surface Translocation by Porphyromonas gingivalis. J Bacteriol.

[108] Fan R, Zhou Y, Chen X, Zhong X, He F, Peng W (2023). Porphyromonas gingivalis Outer Membrane Vesicles Promote Apoptosis via msRNA-Regulated DNA Methylation in Periodontitis. Microbiol Spectr.

[109] Swanson KV, Deng M, Ting JP (2019). The NLRP3 inflammasome: molecular activation and regulation to therapeutics. Nat Rev Immunol.

[110] Ganesh V, Bodewits K, Bartholdson SJ, Natale D, Campopiano DJ, Mareque-Rivas JC (2009). Effective binding and sensing of lipopolysaccharide: combining complementary pattern recognition receptors. Angew Chem Int Ed Engl.

[111] Cecil JD, Sirisaengtaksin N, O'Brien-Simpson NM, Krachler AM (2019). Outer Membrane Vesicle-Host Cell Interactions. Microbiol Spectr.

[112] Cecil JD, O'Brien-Simpson NM, Lenzo JC, Holden JA, Singleton W, Perez-Gonzalez A (2017). Outer Membrane Vesicles Prime and Activate Macrophage Inflammasomes and Cytokine Secretion In Vitro and In Vivo. Front Immunol.

[113] Cecil JD, O'Brien-Simpson NM, Lenzo JC, Holden JA, Chen YY, Singleton W (2016). Differential Responses of Pattern Recognition Receptors to Outer Membrane Vesicles of Three Periodontal Pathogens. PLoS One.

[114] Rocha FG, Ottenberg G, Eure ZG, Davey ME, Gibson FC 3rd (2021). Sphingolipid-Containing Outer Membrane Vesicles Serve as a Delivery Vehicle To Limit Macrophage Immune Response to Porphyromonas gingivalis. Infect Immun.

[115] Sun Y, Shu R, Li CL, Zhang MZ (2010). Gram-negative periodontal bacteria induce the activation of Toll-like receptors 2 and 4, and cytokine production in human periodontal ligament cells. J Periodontol.

[116] Ukai T, Yumoto H, Gibson FC 3rd, Genco CA (2008). Macrophage-elicited osteoclastogenesis in response to bacterial stimulation requires Toll-like receptor 2-dependent tumor necrosis factor-alpha production. Infect Immun.

[117] Shaik-Dasthagirisaheb YB, Huang N, Weinberg EO, Shen SS, Genco CA, Gibson FC 3rd (2015). Aging and contribution of MyD88 and TRIF to expression of TLR pathway-associated genes following stimulation with Porphyromonas gingivalis. J Periodontal Res.

[118] Papadopoulos G, Weinberg EO, Massari P, Gibson FC 3rd, Wetzler LM, Morgan EF (2013). Macrophage-specific TLR2 signaling mediates pathogen-induced TNF-dependent inflammatory oral bone loss. J Immunol.

[119] Wallet SM, Puri V, Gibson FC (2018). Linkage of Infection to Adverse Systemic Complications: Periodontal Disease, Toll-Like Receptors, and Other Pattern Recognition Systems. Vaccines (Basel).

[120] Nichols FC, Levinbook H, Shnaydman M, Goldschmidt J (2001). Prostaglandin E2 secretion from gingival fibroblasts treated with interleukin-1beta: effects of lipid extracts from Porphyromonas gingivalis or calculus. J Periodontal Res.

[121] Nichols FC, Housley WJ, O'Conor CA, Manning T, Wu S, Clark RB (2009). Unique lipids from a common human bacterium represent a new class of Toll-like receptor 2 ligands capable of enhancing autoimmunity. Am J Pathol.

[122] Grenier D (2013). Porphyromonas gingivalis Outer Membrane Vesicles Mediate Coaggregation and Piggybacking of Treponema denticola and Lachnoanaerobaculum saburreum. Int J Dent.

[123] Zhang Z, Liu S, Zhang S, Li Y, Shi X, Liu D (2022). Porphyromonas gingivalis outer membrane vesicles inhibit the invasion of Fusobacterium nucleatum into oral epithelial cells by downregulating FadA and FomA. J Periodontol.

[124] Jung YJ, Jun HK, Choi BK (2017). Porphyromonas gingivalis suppresses invasion of Fusobacterium nucleatum into gingival epithelial cells. J Oral Microbiol.

[125] Grenier D, Bélanger M (1991). Protective effect of Porphyromonas gingivalis outer membrane vesicles against bactericidal activity of human serum. Infect Immun.

[126] Sharma A (2010). Virulence mechanisms of Tannerella forsythia. Periodontol 2000.

[127] Holt SC, Ebersole JL (2005). Porphyromonas gingivalis, Treponema denticola, and Tannerella forsythia: the "red complex", a prototype polybacterial pathogenic consortium in periodontitis. Periodontol 2000.

[128] Friedrich V, Gruber C, Nimeth I, Pabinger S, Sekot G, Posch G (2015). Outer membrane vesicles of Tannerella forsythia: biogenesis, composition, and virulence. Mol Oral Microbiol.

[129] Braun ML, Tomek MB, Grünwald-Gruber C, Nguyen PQ, Bloch S, Potempa JS (2022). Shut-Down of Type IX Protein Secretion Alters the Host Immune Response to Tannerella forsythia and Porphyromonas gingivalis. Front Cell Infect Microbiol.

[130] Tomek MB, Neumann L, Nimeth I, Koerdt A, Andesner P, Messner P (2014). The S-layer proteins of Tannerella forsythia are secreted via a type IX secretion system that is decoupled from protein O-glycosylation. Mol Oral Microbiol.

[131] Narita Y, Sato K, Yukitake H, Shoji M, Nakane D, Nagano K (2014). Lack of a surface layer in Tannerella forsythia mutants deficient in the type IX secretion system. Microbiology (Reading).

[132] Amano A, Chen C, Honma K, Li C, Settem RP, Sharma A (2014). Genetic characteristics and pathogenic mechanisms of periodontal pathogens. Adv Dent Res.

[133] Veith PD, Scott NE, Reynolds EC (2021). Characterization of the O-Glycoproteome of Tannerella forsythia. mSphere.

[134] Socransky SS, Haffajee AD, Cugini MA, Smith C, Kent RL Jr (1998). Microbial complexes in subgingival plaque. J Clin Periodontol.

[135] Lamont RJ, Jenkinson HF (1998). Life below the gum line: pathogenic mechanisms of Porphyromonas gingivalis. Microbiol Mol Biol Rev.

[136] Rosen G, Naor R, Rahamim E, Yishai R, Sela MN (1995). Proteases of Treponema denticola outer sheath and extracellular vesicles. Infect Immun.

[137] Caimano MJ, Bourell KW, Bannister TD, Cox DL, Radolf JD (1999). The Treponema denticola major sheath protein is predominantly periplasmic and has only limited surface exposure. Infect Immun.

[138] Nussbaum G, Ben-Adi S, Genzler T, Sela M, Rosen G (2009). Involvement of Toll-like receptors 2 and 4 in the innate immune response to Treponema denticola and its outer sheath components. Infect Immun.

[139] Jones MM, Vanyo ST, Visser MB (2019). The Msp Protein of Treponema denticola Interrupts Activity of Phosphoinositide Processing in Neutrophils. Infect Immun.

[140] Huang Y, Ni S (2023). Aggregatibacter Actinomycetemcomitans With Periodontitis and Rheumatoid Arthritis. Int Dent J.

[141] Singh AN, Nice JB, Brown AC, Wittenberg NJ (2023). Identifying size-dependent toxin sorting in bacterial outer membrane vesicles. bioRxiv.

[142] Belibasakis GN, Maula T, Bao K, Lindholm M, Bostanci N, Oscarsson J (2019). Virulence and Pathogenicity Properties of Aggregatibacter actinomycetemcomitans. Pathogens.

[143] Ristow LC, Welch RA (2019). RTX Toxins Ambush Immunity's First Cellular Responders. Toxins (Basel).

[144] Taieb F, Petit C, Nougayrède JP, Oswald E (2016). The Enterobacterial Genotoxins: Cytolethal Distending Toxin and Colibactin. EcoSal Plus.

[145] Nakao R, Hirayama S, Yamaguchi T, Senpuku H, Hasegawa H, Suzuki T (2023). A bivalent outer membrane vesicle-based intranasal vaccine to prevent infection of periodontopathic bacteria. Vaccine.

[146] Krueger E, Brown AC (2020). Aggregatibacter actinomycetemcomitans leukotoxin: From mechanism to targeted anti-toxin therapeutics. Mol Oral Microbiol.

[147] Kelk P, Johansson A, Claesson R, Hänström L, Kalfas S (2003). Caspase 1 involvement in human monocyte lysis induced by Actinobacillus actinomycetemcomitans leukotoxin. Infect Immun.

[148] Vega BA, Belinka BA Jr, Kachlany SC (2019). Aggregatibacter actinomycetemcomitans Leukotoxin (LtxA; Leukothera(®)): Mechanisms of Action and Therapeutic Applications. Toxins (Basel).

[149] Kato S, Kowashi Y, Demuth DR (2002). Outer membrane-like vesicles secreted by Actinobacillus actinomycetemcomitans are enriched in leukotoxin. Microb Pathog.

[150] Prince DJ, Patel D, Kachlany SC (2021). Leukotoxin (LtxA/Leukothera) induces ATP expulsion via pannexin-1 channels and subsequent cell death in malignant lymphocytes. Sci Rep.

[151] Thay B, Damm A, Kufer TA, Wai SN, Oscarsson J (2014). Aggregatibacter actinomycetemcomitans outer membrane vesicles are internalized in human host cells and trigger NOD1- and NOD2-dependent NF-κB activation. Infect Immun.

[152] Kieselbach T, Zijnge V, Granström E, Oscarsson J (2015). Proteomics of Aggregatibacter actinomycetemcomitans Outer Membrane Vesicles. PLoS One.

[153] Kieselbach T, Oscarsson J (2017). Dataset of the proteome of purified outer membrane vesicles from the human pathogen Aggregatibacter actinomycetemcomintans. Data Brief.

[154] Iino Y, Hopps RM (1984). The bone-resorbing activities in tissue culture of lipopolysaccharides from the bacteria Actinobacillus actinomycetemcomitans, Bacteroides gingivalis and Capnocytophaga ochracea isolated from human mouths. Arch Oral Biol.

[155] Reddi K, Meghji S, Wilson M, Henderson B (1995). Comparison of the osteolytic activity of surface-associated proteins of bacteria implicated in periodontal disease. Oral Dis.

[156] Han EC, Choi SY, Lee Y, Park JW, Hong SH, Lee HJ (2019). Extracellular RNAs in periodontopathogenic outer membrane vesicles promote TNF-α production in human macrophages and cross the blood-brain barrier in mice. Faseb j.

[157] Abreu AG, Barbosa AS (2017). How Escherichia coli Circumvent Complement-Mediated Killing. Front Immunol.

[158] Berends ET, Kuipers A, Ravesloot MM, Urbanus RT, Rooijakkers SH (2014). Bacteria under stress by complement and coagulation. FEMS Microbiol Rev.

[159] Oscarsson J, Claesson R, Lindholm M, Höglund Åberg C, Johansson A (2019). Tools of Aggregatibacter actinomycetemcomitans to Evade the Host Response. J Clin Med.

[160] Sundqvist G, Johansson E (1982). Bactericidal effect of pooled human serum on Bacteroides melaninogenicus, Bacteroides asaccharolyticus and Actinobacillus actinomycetemcomitans. Scand J Dent Res.

[161] Lindholm M, Metsäniitty M, Granström E, Oscarsson J (2020). Outer membrane vesicle-mediated serum protection in Aggregatibacter actinomycetemcomitans. J Oral Microbiol.

[162] Lamont RJ, Koo H, Hajishengallis G (2018). The oral microbiota: dynamic communities and host interactions. Nat Rev Microbiol.

[163] Brennan CA, Garrett WS (2019). Fusobacterium nucleatum - symbiont, opportunist and oncobacterium. Nat Rev Microbiol.

[164] Engevik MA, Danhof HA, Ruan W, Engevik AC, Chang-Graham AL, Engevik KA (2021). Fusobacterium nucleatum Secretes Outer Membrane Vesicles and Promotes Intestinal Inflammation. mBio.

[165] Chen G, Sun Q, Cai Q, Zhou H (2022). Outer Membrane Vesicles From Fusobacterium nucleatum Switch M0-Like Macrophages Toward the M1 Phenotype to Destroy Periodontal Tissues in Mice. Front Microbiol.

[166] Yang CL, Yang M, Wang YF, Song CC, Du GH, Tang GY (2023). [Effects of Fusobacterium nucleus derived outer membrane vesicles on claudin-4 expression in oral epithelial cells]. Shanghai Kou Qiang Yi Xue.

[167] Idate U, Bhat K, Kulkarni RD, Kotrashetti V, Kugaji M, Kumbar V (2022). Comparison of culture and polymerase chain reaction-restriction fragment length polymorphism for identification of various Capnocytophaga species from subgingival plaque samples of healthy and periodontally diseased individuals. J Oral Maxillofac Pathol.

[168] Naradasu D, Miran W, Sharma S, Takenawa S, Soma T, Nomura N (2021). Biogenesis of Outer Membrane Vesicles Concentrates the Unsaturated Fatty Acid of Phosphatidylinositol in Capnocytophaga ochracea. Front Microbiol.

[169] Zhang S, Miran W, Naradasu D, Guo S, Okamoto A (2020). A Human Pathogen Capnocytophaga Ochracea Exhibits Current Producing Capability. Electrochemistry.

[170] Yin Z, Liu Y, Anniwaer A, You Y, Guo J, Tang Y (2023). Rational Designs of Biomaterials for Combating Oral Biofilm Infections. Adv Mater.

[171] Darveau RP, Curtis MA (2021). Oral biofilms revisited: A novel host tissue of bacteriological origin. Periodontol 2000.

[172] Philip N, Suneja B, Walsh LJ (2018). Ecological Approaches to Dental Caries Prevention: Paradigm Shift or Shibboleth?. Caries Res.

[173] Costalonga M, Herzberg MC (2014). The oral microbiome and the immunobiology of periodontal disease and caries. Immunol Lett.

[174] Curtis MA, Diaz PI, Van Dyke TE (2020). The role of the microbiota in periodontal disease. Periodontol 2000.

[175] Kinane DF, Stathopoulou PG, Papapanou PN (2017). Periodontal diseases. Nat Rev Dis Primers.

[176] Tabatabaei F, Moharamzadeh K, Tayebi L (2020). Three-Dimensional In Vitro Oral Mucosa Models of Fungal and Bacterial Infections. Tissue Eng Part B Rev.

[177] Wang L, Ganly I (2014). The oral microbiome and oral cancer. Clin Lab Med.

[178] Chowdhry A, Kapoor P, Bhargava D, Bagga DK (2023). Exploring the oral microbiome: an updated multidisciplinary oral healthcare perspective. Discoveries (Craiova).

[179] Hajishengallis G, Lamont RJ, Koo H (2023). Oral polymicrobial communities: Assembly, function, and impact on diseases. Cell Host Microbe.

[180] Papapanou PN, Sanz M, Buduneli N, Dietrich T, Feres M, Fine DH (2018). Periodontitis: Consensus report of workgroup 2 of the 2017 World Workshop on the Classification of Periodontal and Peri-Implant Diseases and Conditions. J Periodontol.

[181] Meyle J, Chapple I (2015). Molecular aspects of the pathogenesis of periodontitis. Periodontol 2000.

[182] Darveau RP, Tanner A, Page RC (1997). The microbial challenge in periodontitis. Periodontol 2000.

[183] Mohanty R, Asopa SJ, Joseph MD, Singh B, Rajguru JP, Saidath K (2019). Red complex: Polymicrobial conglomerate in oral flora: A review. J Family Med Prim Care.

[184] Sulistiowati CP, Suhartono M, Rahmawati DF, Ulfah N, Supandi SK, Wijaksana IKE (2023). In-Vitro Inhibitory Efficacy of 3 Types of Probiotics on the Growth of Aggregatibacter actinomycetemcomitans Bacteria. Front Biosci (Landmark Ed).

[185] Lee S, Haraga H, Satoh T, Mutoh N, Watanabe K, Hamada N (2024). Effect of periodontitis induced by Fusobacterium nucleatum on the microbiota of the gut and surrounding organs. Odontology.

[186] Bartruff JB, Yukna RA, Layman DL (2005). Outer membrane vesicles from Porphyromonas gingivalis affect the growth and function of cultured human gingival fibroblasts and umbilical vein endothelial cells. J Periodontol.

[187] Diaz PI, Rogers AH (2004). The effect of oxygen on the growth and physiology of Porphyromonas gingivalis. Oral Microbiol Immunol.

[188] O'Brien-Simpson NM, Pathirana RD, Walker GD, Reynolds EC (2009). Porphyromonas gingivalis RgpA-Kgp proteinase-adhesin complexes penetrate gingival tissue and induce proinflammatory cytokines or apoptosis in a concentration-dependent manner. Infect Immun.

[189] Ciaston I, Budziaszek J, Satala D, Potempa B, Fuchs A, Rapala-Kozik M (2022). Proteolytic Activity-Independent Activation of the Immune Response by Gingipains from Porphyromonas gingivalis. mBio.

[190] Fitzpatrick RE, Aprico A, Wijeyewickrema LC, Pagel CN, Wong DM, Potempa J (2009). High molecular weight gingipains from Porphyromonas gingivalis induce cytokine responses from human macrophage-like cells via a nonproteolytic mechanism. J Innate Immun.

[191] Nowakowska Z, Madej M, Grad S, Wang T, Hackett M, Miller DP (2021). Phosphorylation of major Porphyromonas gingivalis virulence factors is crucial for their processing and secretion. Mol Oral Microbiol.

[192] Wee P, Wang Z (2017). Epidermal Growth Factor Receptor Cell Proliferation Signaling Pathways. Cancers (Basel).

[193] Pendaries C, Tronchère H, Arbibe L, Mounier J, Gozani O, Cantley L (2006). PtdIns5P activates the host cell PI3-kinase/Akt pathway during Shigella flexneri infection. Embo j.

[194] Li L, Michel R, Cohen J, Decarlo A, Kozarov E (2008). Intracellular survival and vascular cell-to-cell transmission of Porphyromonas gingivalis. BMC Microbiol.

[195] Xia Q, Wang T, Taub F, Park Y, Capestany CA, Lamont RJ (2007). Quantitative proteomics of intracellular Porphyromonas gingivalis. Proteomics.

[196] Jain S, Darveau RP (2010). Contribution of Porphyromonas gingivalis lipopolysaccharide to periodontitis. Periodontol 2000.

[197] Rosen G, Naor R, Kutner S, Sela MN (1994). Characterization of fibrinolytic activities of Treponema denticola. Infect Immun.

[198] Silva LM, Divaris K, Bugge TH, Moutsopoulos NM (2023). Plasmin-Mediated Fibrinolysis in Periodontitis Pathogenesis. J Dent Res.

[199] Wikström MB, Dahlén G, Linde A (1983). Fibrinogenolytic and fibrinolytic activity in oral microorganisms. J Clin Microbiol.

[200] Sochaj-Gregorczyk A, Ksiazek M, Waligorska I, Straczek A, Benedyk M, Mizgalska D (2020). Plasmin inhibition by bacterial serpin: Implications in gum disease. Faseb j.

[201] Grenier D (2013). Binding properties of Treponema denticola lipooligosaccharide. J Oral Microbiol.

[202] Kademani D (2007). Oral cancer. Mayo Clin Proc.

[203] Chai AWY, Lim KP, Cheong SC (2020). Translational genomics and recent advances in oral squamous cell carcinoma. Semin Cancer Biol.

[204] Galeano Niño JL, Wu H, LaCourse KD, Kempchinsky AG, Baryiames A, Barber B (2022). Effect of the intratumoral microbiota on spatial and cellular heterogeneity in cancer. Nature.

[205] Yang L, Li A, Wang Y, Zhang Y (2023). Intratumoral microbiota: roles in cancer initiation, development and therapeutic efficacy. Signal Transduct Target Ther.

[206] Li R, Xiao L, Gong T, Liu J, Li Y, Zhou X (2023). Role of oral microbiome in oral oncogenesis, tumor progression, and metastasis. Mol Oral Microbiol.

[207] Chang C, Geng F, Shi X, Li Y, Zhang X, Zhao X (2019). The prevalence rate of periodontal pathogens and its association with oral squamous cell carcinoma. Appl Microbiol Biotechnol.

[208] Su SC, Chang LC, Huang HD, Peng CY, Chuang CY, Chen YT (2021). Oral microbial dysbiosis and its performance in predicting oral cancer. Carcinogenesis.

[209] Groeger S, Herrmann JM, Chakraborty T, Domann E, Ruf S, Meyle J (2022). Porphyromonas gingivalis W83 Membrane Components Induce Distinct Profiles of Metabolic Genes in Oral Squamous Carcinoma Cells. Int J Mol Sci.

[210] Chang C, Wang H, Liu J, Pan C, Zhang D, Li X (2019). Porphyromonas gingivalis Infection Promoted the Proliferation of Oral Squamous Cell Carcinoma Cells through the miR-21/PDCD4/AP-1 Negative Signaling Pathway. ACS Infect Dis.

[211] Zheng X, Liu R, Zhou C, Yu H, Luo W, Zhu J (2021). ANGPTL4-Mediated Promotion of Glycolysis Facilitates the Colonization of Fusobacterium nucleatum in Colorectal Cancer. Cancer Res.

[212] Peng RT, Sun Y, Zhou XD, Liu SY, Han Q, Cheng L (2022). Treponema denticola Promotes OSCC Development via the TGF-β Signaling Pathway. J Dent Res.

[213] Abdulkareem AA, Shelton RM, Landini G, Cooper PR, Milward MR (2018). Periodontal pathogens promote epithelial-mesenchymal transition in oral squamous carcinoma cells in vitro. Cell Adh Migr.

[214] Kamarajan P, Ateia I, Shin JM, Fenno JC, Le C, Zhan L (2020). Periodontal pathogens promote cancer aggressivity via TLR/MyD88 triggered activation of Integrin/FAK signaling that is therapeutically reversible by a probiotic bacteriocin. PLoS Pathog.

[215] Tang B, Wang K, Jia YP, Zhu P, Fang Y, Zhang ZJ (2016). Fusobacterium nucleatum-Induced Impairment of Autophagic Flux Enhances the Expression of Proinflammatory Cytokines via ROS in Caco-2 Cells. PLoS One.

[216] Liu S, Zhou X, Peng X, Li M, Ren B, Cheng G (2020). Porphyromonas gingivalis Promotes Immunoevasion of Oral Cancer by Protecting Cancer from Macrophage Attack. J Immunol.

[217] Liu D, Liu S, Liu J, Miao L, Zhang S, Pan Y (2021). sRNA23392 packaged by Porphyromonas gingivalis outer membrane vesicles promotes oral squamous cell carcinomas migration and invasion by targeting desmocollin-2. Mol Oral Microbiol.

[218] Okegawa T, Pong RC, Li Y, Hsieh JT (2004). The role of cell adhesion molecule in cancer progression and its application in cancer therapy. Acta Biochim Pol.

[219] Dusek RL, Attardi LD (2011). Desmosomes: new perpetrators in tumour suppression. Nat Rev Cancer.

[220] Li K, Wu R, Zhou M, Tong H, Luo KQ (2021). Desmosomal proteins of DSC2 and PKP1 promote cancer cells survival and metastasis by increasing cluster formation in circulatory system. Sci Adv.

[221] Huang Y, Hong W, Wei X (2022). The molecular mechanisms and therapeutic strategies of EMT in tumor progression and metastasis. J Hematol Oncol.

[222] Thiery JP, Acloque H, Huang RY, Nieto MA (2009). Epithelial-mesenchymal transitions in development and disease. Cell.

[223] Gong T, Chen Q, Mao H, Zhang Y, Ren H, Xu M (2022). Outer membrane vesicles of Porphyromonas gingivalis trigger NLRP3 inflammasome and induce neuroinflammation, tau phosphorylation, and memory dysfunction in mice. Front Cell Infect Microbiol.

[224] Farrugia C, Stafford GP, Murdoch C (2020). Porphyromonas gingivalis Outer Membrane Vesicles Increase Vascular Permeability. J Dent Res.

[225] Privratsky JR, Newman PJ (2014). PECAM-1: regulator of endothelial junctional integrity. Cell Tissue Res.

[226] Chistiakov DA, Orekhov AN, Bobryshev YV (2015). Endothelial Barrier and Its Abnormalities in Cardiovascular Disease. Front Physiol.

[227] Ishikawa M, Yoshida K, Okamura H, Ochiai K, Takamura H, Fujiwara N (2013). Oral Porphyromonas gingivalis translocates to the liver and regulates hepatic glycogen synthesis through the Akt/GSK-3β signaling pathway. Biochim Biophys Acta.

[228] Takamura H, Yoshida K, Okamura H, Fujiwara N, Ozaki K (2016). Porphyromonas gingivalis attenuates the insulin-induced phosphorylation and translocation of forkhead box protein O1 in human hepatocytes. Arch Oral Biol.

[229] Konig MF, Paracha AS, Moni M, Bingham CO 3rd, Andrade F (2015). Defining the role of Porphyromonas gingivalis peptidylarginine deiminase (PPAD) in rheumatoid arthritis through the study of PPAD biology. Ann Rheum Dis.

[230] Ma X, Shin YJ, Yoo JW, Park HS, Kim DH (2023). Extracellular vesicles derived from Porphyromonas gingivalis induce trigeminal nerve-mediated cognitive impairment. J Adv Res.

[231] Raineri EJM, Altulea D, van Dijl JM (2022). Staphylococcal trafficking and infection-from 'nose to gut' and back. FEMS Microbiol Rev.

[232] Courjon J, Munro P, Benito Y, Visvikis O, Bouchiat C, Boyer L (2015). EDIN-B Promotes the Translocation of Staphylococcus aureus to the Bloodstream in the Course of Pneumonia. Toxins (Basel).

[233] Pisani F, Pisani V, Arcangeli F, Harding A, Singhrao SK (2023). Treponema denticola Has the Potential to Cause Neurodegeneration in the Midbrain via the Periodontal Route of Infection-Narrative Review. Int J Environ Res Public Health.

[234] Abisado RG, Benomar S, Klaus JR, Dandekar AA, Chandler JR (2018). Erratum for Abisado et al. "Bacterial Quorum Sensing and Microbial Community Interactions". mBio.

[235] An SJ, Ha KW, Jun HK, Kim HY, Choi BK (2023). Reduced proinflammatory activity of outer membrane vesicles of Tannerella forsythia treated with quorum sensing inhibitors. Mol Oral Microbiol.

[236] Swartz MA (2001). The physiology of the lymphatic system. Adv Drug Deliv Rev.

[237] Xiang SD, Scholzen A, Minigo G, David C, Apostolopoulos V, Mottram PL (2006). Pathogen recognition and development of particulate vaccines: does size matter?. Methods.

[238] Cubas R, Zhang S, Kwon S, Sevick-Muraca EM, Li M, Chen C (2009). Virus-like particle (VLP) lymphatic trafficking and immune response generation after immunization by different routes. J Immunother.

[239] Dane KY, Nembrini C, Tomei AA, Eby JK, O'Neil CP, Velluto D (2011). Nano-sized drug-loaded micelles deliver payload to lymph node immune cells and prolong allograft survival. J Control Release.

[240] Fukanoki S, Matsumoto K, Mori H, Takeda R (2000). Relation between antigen release and immune response of oil adjuvanted vaccines in chickens. J Vet Med Sci.

[241] Bai D, Nakao R, Ito A, Uematsu H, Senpuku H (2015). Immunoreactive antigens recognized in serum samples from mice intranasally immunized with Porphyromonas gingivalis outer membrane vesicles. Pathog Dis.

[242] Tamura S, Tanimoto T, Kurata T (2005). Mechanisms of broad cross-protection provided by influenza virus infection and their application to vaccines. Jpn J Infect Dis.

[243] Veith PD, Glew MD, Gorasia DG, Chen D, O'Brien-Simpson NM, Reynolds EC (2019). Localization of Outer Membrane Proteins in Treponema denticola by Quantitative Proteome Analyses of Outer Membrane Vesicles and Cellular Fractions. J Proteome Res.

[244] Huang J, Wu Z, Xu J (2022). Effects of Biofilm Nano-Composite Drugs OMVs-MSN-5-FU on Cervical Lymph Node Metastases From Oral Squamous Cell Carcinoma. Front Oncol.

[245] André T, Boni C, Mounedji-Boudiaf L, Navarro M, Tabernero J, Hickish T (2004). Oxaliplatin, fluorouracil, and leucovorin as adjuvant treatment for colon cancer. N Engl J Med.

[246] Vazquez T, Florez-White M (2020). A patient with squamous cell carcinoma in-situ successfully treated with intralesional 5-Fluorouracil and topical trichloroacetic acid. J Dermatolog Treat.

[247] Longley DB, Harkin DP, Johnston PG (2003). 5-fluorouracil: mechanisms of action and clinical strategies. Nat Rev Cancer.

[248] Nagata M, Nakayama H, Tanaka T, Yoshida R, Yoshitake Y, Fukuma D (2011). Overexpression of cIAP2 contributes to 5-FU resistance and a poor prognosis in oral squamous cell carcinoma. Br J Cancer.

[249] Kim SM, Ha E, Kim J, Cho C, Shin SJ, Seo JH (2020). NAA10 as a New Prognostic Marker for Cancer Progression. Int J Mol Sci.

[250] Le MK, Vuong HG, Nguyen TTT, Kondo T (2023). NAA10 overexpression dictates distinct epigenetic, genetic, and clinicopathological characteristics in adult gliomas. J Neuropathol Exp Neurol.

[251] Zhang H, Shan Y, Dong L (2014). A comparison of TiO2 and ZnO nanoparticles as photosensitizers in photodynamic therapy for cancer. J Biomed Nanotechnol.

[252] Zhou JY, Wang WJ, Zhang CY, Ling YY, Hong XJ, Su Q (2022). Ru(II)-modified TiO(2) nanoparticles for hypoxia-adaptive photo-immunotherapy of oral squamous cell carcinoma. Biomaterials.

[253] Yaremko ZM, Tkachenko NH, Bellmann C, Pich A (2006). Redispergation of TiO2 particles in aqueous solutions. J Colloid Interface Sci.

[254] Kan SA, Zhang LW, Wang YC, Chiang CY, Chen MH, Huang SH (2024). Bacterial Outer Membrane Vesicle (OMV)-Encapsulated TiO(2) Nanoparticles: A Dual-Action Strategy for Enhanced Radiotherapy and Immunomodulation in Oral Cancer Treatment. Nanomaterials (Basel).

[255] Ries J, Vairaktaris E, Mollaoglu N, Wiltfang J, Neukam FW, Nkenke E (2008). Expression of melanoma-associated antigens in oral squamous cell carcinoma. J Oral Pathol Med.

[256] Shimizu M, Imai M (2008). Effect of the antibody immunotherapy by the anti-MUC1 monoclonal antibody to the oral squamous cell carcinoma in vitro. Biol Pharm Bull.

[257] Ning X, Zheng H, Tu Y, Guo Q, Ren B, Wu L (2025). Branched-chain amino acids promote gelatinase secretion from human periodontal ligament stem cells through nuclear factor kappa-B signaling. Arch Oral Biol.

[258] Zheng H, Tu Y, Ning X, Guo Q, Ren B, Xie J (2025). The microbial metabolite isovaleric acid aggravates gelatinase-mediated periodontal tissue destruction via the NF-κB signaling pathway. J Periodontol.

[259] de Abreu RC, Fernandes H, da Costa Martins PA, Sahoo S, Emanueli C, Ferreira L (2020). Native and bioengineered extracellular vesicles for cardiovascular therapeutics. Nat Rev Cardiol.

[260] Mirlashari MR, Høiby EA, Holst J, Lyberg T (2001). Outer membrane vesicles from Neisseria meningitidis: effects on cytokine production in human whole blood. Cytokine.

[261] Bai X, Li C, Qiu J, Wu L, Liu X, Yin T (2025). A "plug-and-display" nanoparticle based on attenuated outer membrane vesicles enhances the immunogenicity of protein antigens. J Control Release.

[262] Kim OY, Hong BS, Park KS, Yoon YJ, Choi SJ, Lee WH (2013). Immunization with Escherichia coli outer membrane vesicles protects bacteria-induced lethality via Th1 and Th17 cell responses. J Immunol.

[263] Gnopo YMD, Misra A, Hsu HL, DeLisa MP, Daniel S, Putnam D (2020). Induced fusion and aggregation of bacterial outer membrane vesicles: Experimental and theoretical analysis. J Colloid Interface Sci.

[264] Weyant KB, Oloyede A, Pal S, Liao J, Jesus MR, Jaroentomeechai T (2023). A modular vaccine platform enabled by decoration of bacterial outer membrane vesicles with biotinylated antigens. Nat Commun.

[265] Gerritzen MJH, Martens DE, Wijffels RH, van der Pol L, Stork M (2017). Bioengineering bacterial outer membrane vesicles as vaccine platform. Biotechnol Adv.

[266] Zanella I, König E, Tomasi M, Gagliardi A, Frattini L, Fantappiè L (2021). Proteome-minimized outer membrane vesicles from Escherichia coli as a generalized vaccine platform. J Extracell Vesicles.

[267] Buddenborg C, Daudel D, Liebrecht S, Greune L, Humberg V, Schmidt MA (2008). Development of a tripartite vector system for live oral immunization using a gram-negative probiotic carrier. Int J Med Microbiol.

